# A new mouse model of Ehlers-Danlos syndrome generated using CRISPR/Cas9-mediated genomic editing

**DOI:** 10.1242/dmm.048963

**Published:** 2021-12-23

**Authors:** Yuko Nitahara-Kasahara, Shuji Mizumoto, Yukiko U. Inoue, Shota Saka, Guillermo Posadas-Herrera, Aki Nakamura-Takahashi, Yuki Takahashi, Ayana Hashimoto, Kohei Konishi, Shinji Miyata, Chiaki Masuda, Emi Matsumoto, Yasunobu Maruoka, Takahiro Yoshizawa, Toshiki Tanase, Takayoshi Inoue, Shuhei Yamada, Yoshihiro Nomura, Shin'ichi Takeda, Atsushi Watanabe, Tomoki Kosho, Takashi Okada

**Affiliations:** 1 Department of Biochemistry and Molecular Biology, Nippon Medical School, Tokyo 113-8603, Japan; 2 Division of Molecular and Medical Genetics, Center for Gene and Cell Therapy, The Institute of Medical Science, The University of Tokyo, Tokyo 108-8639, Japan; 3 Department of Pathobiochemistry, Faculty of Pharmacy, Meijo University, Nagoya 468-8503, Japan; 4 Department of Biochemistry and Cellular Biology, National Institute of Neuroscience, National Center of Neurology and Psychiatry, Kodaira 187-8502, Japan; 5 Faculty of Agriculture, Tokyo University of Agriculture and Technology, Fuchu 183-8509, Japan; 6 Department of Molecular Therapy, National Institute of Neuroscience, National Center of Neurology and Psychiatry, Kodaira 187-8502, Japan; 7 Department of Pharmacology, Tokyo Dental College, Tokyo 101-0061, Japan; 8 Department of Medical Genetics, Shinshu University School of Medicine, Matsumoto 390-8621, Japan; 9 Division of Animal Research, Research Center for Supports to Advanced Science, Shinshu University, Matsumoto 390-8621, Japan; 10 Department of Pediatric Dentistry, Tokyo Dental College, Tokyo 101-0061, Japan; 11 Division of Clinical Genetics, Kanazawa University Hospital, Kanazawa 920-8640, Japan; 12 Center for Medical Genetics, Shinshu University Hospital, Matsumoto 390-8621, Japan; 13 Research Center for Supports to Advanced Science, Shinshu University, Matsumoto 390-8621, Japan; 14 Division of Clinical Sequencing, Shinshu University School of Medicine, Matsumoto 390-8621, Japan

**Keywords:** Musculocontractural Ehlers-Danlos syndrome, Dermatan sulfate, Mouse model, CRISPR/Cas9, Myopathy

## Abstract

Musculocontractural Ehlers-Danlos syndrome (mcEDS) is caused by generalized depletion of dermatan sulfate (DS) due to biallelic pathogenic variants in *CHST14* encoding dermatan 4-*O*-sulfotransferase 1 (D4ST1) (mcEDS-*CHST14*). Here, we generated mouse models for mcEDS-*CHST14* carrying homozygous mutations (1 bp deletion or 6 bp insertion/10 bp deletion) in *Chst14* through CRISPR/Cas9 genome engineering to overcome perinatal lethality in conventional *Chst14*-deleted knockout mice. DS depletion was detected in the skeletal muscle of these genome-edited mutant mice, consistent with loss of D4ST1 activity. The mutant mice showed common pathophysiological features, regardless of the variant, including growth impairment and skin fragility. Notably, we identified myopathy-related phenotypes. Muscle histopathology showed variation in fiber size and spread of the muscle interstitium. Decorin localized diffusely in the spread endomysium and perimysium of skeletal muscle, unlike in wild-type mice. The mutant mice showed lower grip strength and decreased exercise capacity compared to wild type, and morphometric evaluation demonstrated thoracic kyphosis in mutant mice. The established CRISPR/Cas9-engineered *Chst14* mutant mice could be a useful model to further our understanding of mcEDS pathophysiology and aid in the development of novel treatment strategies.

## Abstract (precis)

**Summary:** CRISPR/Cas9 genome-engineered *Chst14^−/−^* mouse models of musculocontractural Ehlers-Danlos syndrome (mcEDS) display similar myopathic features (particularly those caused by the loss of D4ST1) to mcEDS patients and may facilitate further understanding of mcEDS.

## INTRODUCTION

Ehlers-Danlos syndrome (EDS) is a clinically and genetically heterogeneous group of heritable connective tissue disorders, the hallmarks of which include joint hypermobility, skin hyperextensibility and tissue fragility (Malfait et al., 2020). Currently, EDS is classified into 14 subtypes based on clinical, molecular and biochemical features according to the 2017 International Classification and more recent updates (Malfait et al., 2020, 2017). The musculocontractural EDS (mcEDS) subtype is caused by defective biosynthesis of dermatan sulfate (DS). Most patients with mcEDS were found to have biallelic pathogenic variants in the gene for carbohydrate sulfotransferase 14 (*CHST14*), encoding dermatan 4-*O*-sulfotransferase 1 (D4ST1) (mcEDS-*CHST14*). The remaining patients were found to have biallelic pathogenic variants in the gene for DS epimerase (*DSE*) (mcEDS-*DSE*) (Brady et al., 2017; Kosho et al., 2020). The mcEDS subtype is clinically characterized by multiple malformations (e.g. craniofacial features, multiple congenital contractures, and ocular and visceral malformations) and progressive connective tissue fragility-related manifestations (e.g. skin hyperextensibility and fragility, joint hypermobility with luxation, progressive spinal and foot deformities, large subcutaneous hematomas and visceral ruptures) (Kosho, 2016; Kosho et al., 2011, 2020). To date, 48 patients from 33 families have been reported to have mcEDS-*CHST14*, and eight patients from six families have been reported with mcEDS-*DSE*.

Myopathy has been observed in patients with mcEDS-*CHST14*. Patients typically show gross motor developmental delay associated with hypotonia. Dundar et al. described a patient who underwent needle electromyography and nerve conduction studies, which revealed reduced amplitude muscle action potentials with normal distal latency time and nerve conduction velocity (Dundar et al., 1997). Voermans et al. described a patient who underwent quantitative muscle ultrasonography, which showed increased echo intensity in the forearm extensors and anterior tibial muscles as well as marked bilateral atrophy of the forearm flexors, forearm extensors and quadriceps (Voermans et al., 2012). The patient also underwent nerve conduction studies showing low compound muscle action potential amplitudes in the distal muscles, and needle electromyography showed an abnormal and mixed pattern of short-duration, low-amplitude, polyphasic motor units, as well as polyphasic motor units with a longer duration and higher amplitude, reflecting an increase in fiber-size diameter. Muscle biopsy demonstrated fiber type 1 predominance without fiber-type grouping, increased variation in the diameter of both type 1 and type 2 fibers, and some type 1 fibers in close proximity to lobulated fibers. Elevated serum creatine kinase (CK) levels were observed in three of five patients whose data were available (Janecke et al., 2016).

D4ST1 is a critical enzyme in the biosynthesis of DS and catalyzes the 4-*O*-sulfation of *N*-acetyl-D-galactosamine (GalNAc) in the sequence L-iduronic acid (IdoA)-GalNAc (Evers et al., 2001; Mikami et al., 2003; Miyake et al., 2010), which occurs immediately after epimerization of D-glucuronic acid (GlcA) to IdoA by DSE and/or DSE2 (Maccarana et al., 2006; Mizumoto and Sugahara, 2012; Pacheco et al., 2009). DSE2 is a homolog of DSE, and its homozygous mutation as well as single-nucleotide polymorphisms cause diaphragmatic hernia and bipolar disorder, respectively (Goossens et al., 2003; Zayed et al., 2010). Decorin is a proteoglycan harboring a single glycosaminoglycan (GAG) chain, which plays an important role in the assembly of collagen fibrils, possibly through an electrostatic interaction between decorin DS chains and adjacent collagen fibrils (Iozzo, 1998; Nomura, 2006). Decorin GAG chains from the skin fibroblasts of patients with mcEDS-*CHST14* contain no DS disaccharides and are completely replaced by chondroitin sulfate (CS), whereas decorin GAG chains from control patients contain mainly DS disaccharides (Miyake et al., 2010). Furthermore, DS disaccharides were not observed in the urine samples from patients with mcEDS-*CHST14* (Mizumoto et al., 2017). Transmission electron microscopy (TEM) of skin specimens from patients with mcEDS-*CHST14* revealed that collagen fibrils in the papillary to reticular dermis were dispersed, in contrast to control specimens, in which collagen fibrils were assembled regularly and tightly (Hirose et al., 2019). Furthermore, Cupromeronic Blue-stained TEM, used to visualize GAG chains, demonstrated that the affected GAG chains were linear, stretching from the outer surface of collagen fibrils to adjacent fibrils, in contrast to the curved GAG chains, maintaining close contact with attached collagen fibrils, in control specimens (Hirose et al., 2019).

A *Chst14*-deleted knockout (KO) mouse model was previously generated through homologous recombination targeting the single coding exon of *Chst14* (Tang et al., 2010), which demonstrated reduced fertility, a kinked tail and increased skin fragility as pathological phenotypes (Bian et al., 2011; Yoshizawa et al., 2018). In addition, *Chst14* KO mice with a mixed C57BL/6J and 129S2/SvImJ genetic background showed placental vascular abnormalities, which could be relevant to vascular events in mcEDS-*CHST14* that result in large subcutaneous hematomas (Yoshizawa et al., 2018), and structural disorganization of the skin collagen fibril network, similar to the findings observed in the skin of patients with mcEDS-*CHST14* (Hirose et al., 2021). However, a systematic and detailed investigation of mouse models for mcEDS, which is critical for the development of novel therapeutic modalities, has not yet been reported.

The *Chst14* KO mice with a C57BL/6J and 129S2/SvImJ mixed background are considered to be affected in their behavioral characteristics (Gurumurthy et al., 2015) because the 129 substrains of mouse perform differently from the C57BL/6J strain in a variety of behavioral tests (Lamacchia et al., 2007). In order to evaluate the motor function more sensitively and accurately than conventional *Chst14* KO, we generated a line of *Chst14* KO mice in the C57BL/6J strain. To overcome perinatal lethality in *Chst14* KO mice (<1%) (Yoshizawa et al., 2018), we established gene-edited mouse strains for *Chst14* using the CRISPR/Cas9 system. The aim of the present study was to comprehensively evaluate pathophysiological phenotypes in the CRISPR/Cas9 genome-engineered mouse as a model for mcEDS-*CHST14* and to compare the pathophysiological phenotypes of this model with those of the *Chst14* KO/C57BL/6J mouse strain. In particular, we focused on myopathy and associated muscle function caused by the loss of D4ST1 because the myopathy phenotype has not been studied extensively.

## RESULTS

### Development of the *Chst14* gene trap-KO strain and CRISPR/Cas9-genome engineered *Chst14^−/−^* mice

**Fig. 1.**
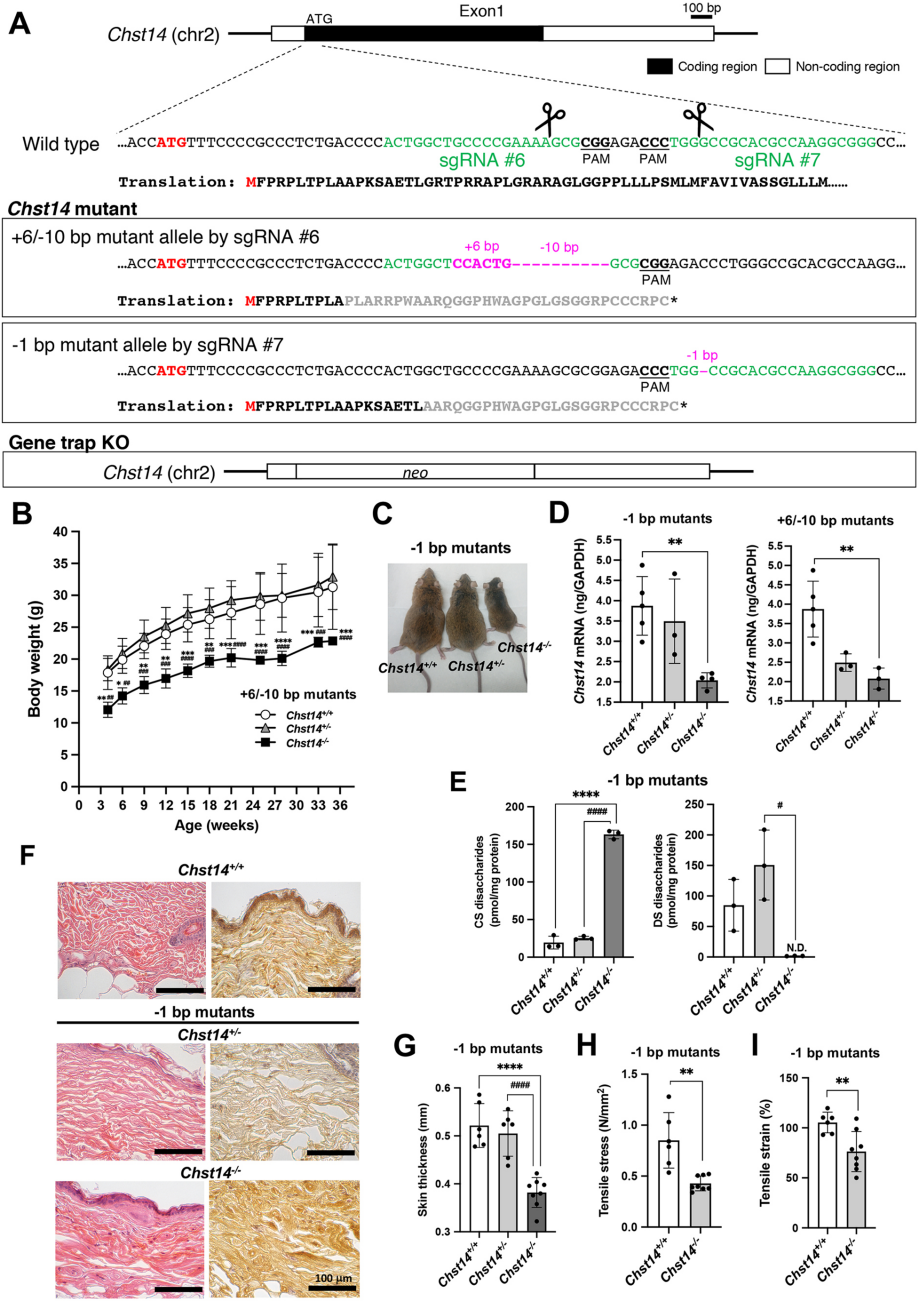
**Generation and characterization of mice carrying mutations in *Chst14* using CRISPR/Cas9 genome engineering.** (A) Targeting strategy for knocking out *Chst14*. The genetic configuration of murine *Chst14* (top). The region downstream from the translation start site ATG (shown in red) is enlarged and the sgRNA sequences (#6 and #7) contiguous to their protospacer adjacent motifs (PAMs) are presented in green. Two mutant alleles selected for further analyses (middle), namely a 6-bp insertion/10-bp deletion (+6/−10-bp mutant) and a 1-bp deletion (−1-bp mutant), are shown in magenta. The translated amino acid sequences are depicted below the corresponding DNA sequences. The reading frame conversions are presented in gray and the newly emerged stop codons are marked with asterisks. *Chst14* gene trap-knockout (KO) mice were created by replacing *Chst14* with the neomycin resistance gene (*neo*) (Tang et al., 2010) (bottom). The *Chst14* KO*/*C57BL/6J mouse strain was created using speed congenic strategy. (B) Growth curve of 4- to 36-week-old wild-type (*Chst14^+/+^*, 3 males and 5 females), +6/−10-bp mutant heterozygous (*Chst14^+/−^*, 5 males and 1 female) and homozygous (*Chst14^−/−^*, 1 male and 4 females) mice. (C) One-year-old *Chst14^+/+^*, *Chst14^+/−^* and *Chst14^−/−^* (−1-bp mutant) mice. (D) Quantification of *Chst14* mRNA in the cardiac muscle from *Chst14^+/+^* (3 males and 2 females), *Chst14^+/−^* (−1-bp mutants, 2 males and 1 female; +6/−10-bp mutants, 1 male and 2 females) and *Chst14^−/−^* (−1-bp mutants, 1 male and 3 females; +6/−10-bp mutants, 2 males and 1 female) mice using real-time PCR. Quantitative data are normalized to glyceraldehyde 3-phosphate dehydrogenase (*Gapdh*) expression level. (E) Total amounts of chondroitin sulfate (CS) and dermatan sulfate (DS) disaccharides derived from the tibialis anterior muscle of 1-year-old *Chst14^+/+^* (1 male and 2 females), −1-bp mutant *Chst14^+/−^* (2 males and 1 female) and *Chst14^−/−^* (1 male and 2 females) mice, analyzed using anion-exchange HPLC following enzymatic digestion. N.D., not detected (<0.1 pmol/mg protein). (F) Hematoxylin and Eosin (H&E) staining (left column) and immunohistochemical analysis using horseradish peroxidase (HRP)-diaminobenzidine (DAB) labeling of decorin (right column) in the skin derived from *Chst14^+/+^*, −1-bp mutant *Chst14^+/−^* and *Chst14^−/−^* mice. Scale bars: 100 µm. (G) Skin thickness of *Chst14^+/+^* (1 male and 2 females), −1-bp mutant *Chst14^+/−^* (2 males and 1 female) and *Chst14^−/−^* (1 male and 3 females) mice. Data were measured twice per mouse. (H,I) Tensile stress (N/mm^2^) (H) and tensile strain (%) (I) in *Chst14^+/+^* (two measurements per mouse for a group of 1 male and 2 females) and −1-bp mutant *Chst14^−/−^* (1 male and 3 females) mice. Data are presented as mean±s.d. Statistical differences, compared to *Chst14^+/+^* (*P<0.05, **P<0.01, ***P<0.001 and ****P<0.0001) and *Chst14^+/−^* (^#^*P*<0.05, ^##^*P*<0.01, ^###^*P*<0.001 and ^####^*P*<0.0001) mice, were evaluated using one- or two-way ANOVA and unpaired two-tailed Student's *t-*test.

Homozygous *Chst14* gene trap-KO/C57BL/6J mice (*Chst14* gene trap-KO) were successfully maintained for five generations using heterozygous breeding pairs, C57BL/6J and 129S2/SvImJ mixed background. In a *Chst14* gene trap-KO strain after F11, >91.4% of 58 markers were replaced by a sequence of C57BL/6J origin, when evaluated by discrimination of simple sequence length polymorphism (SSLP) markers (Table S1) (Lamacchia et al., 2007). However, this was not sufficient to pursue an mcEDS characterization study, and there was a significant reduction in birth rate as the gene replacement rate for C57BL/6J increased (Table S2). To develop a viable *Chst14* mutant mouse model, we designed six single-guide RNAs (sgRNAs) targeting the region downstream from the translation start site (ATG) (Fig. 1A) using the web-based CRISPR design tool, CRISPOR (http://crispor.tefor.net/) (Concordet and Haeussler, 2018). We cloned these sgRNA sequences into the Cas9/sgRNA expression vector, pX330, to evaluate DNA cleavage activities using a previously reported *in vitro* method (Mashiko et al., 2013). sgRNAs #6 and #7 showed much higher cleavage activity than the others (Fig. S1A,B); we selected these two sgRNAs for KO mouse generation. We directly injected the purified pX330 containing sgRNA #6 or #7 into mouse fertilized eggs to introduce DNA double-strand breaks (DSBs), eventually resulting in non-homologous end joining-mediated gene disruptions. As the DNA cleavage efficiency of these sgRNAs *in vivo* was also high, we confirmed that almost all newborns carried various modified alleles (Table S3). Among them, we successfully established two lines of frameshift mutation and introduced a premature termination codon, the 6 bp insertion/10 bp deletion (+6/−10-bp mutant), c. 31_40delinsCCACTG; p.(Ala11Profs*34), by sgRNA #6 and the 1 bp deletion (–1-bp mutant), c. 57delG; p.(Gly19Alafs*26), by sgRNA #7 (Fig. 1A; Fig. S2A), both of which caused the reading frame conversions to generate ectopic stop codons (Fig. 1A). These mutant alleles were maintained as homozygous *Chst14^−/−^* until F4 generation by interbreeding heterozygous mice. To exclude the unintended side effects from possible off-target cleavages, we adopted the following two approaches. First, we predicted the off-target candidate loci for sgRNA #6 and #7 listed in Table S4 by employing CRISPOR (Concordet and Haeussler, 2018) and checked whether the candidate sites were intact using the genomic DNA prepared from the founder KO mice. As a result, we did not detect any signs of off-target cleavage within the genomic regions we evaluated (Fig. S2B). Second, we analyzed two independently generated mutant mouse lines (+6/−10-bp and −1-bp mutants) to confirm that the same results were obtained regardless of the mutant allele, as described below. This consistency ensured that, if by any chance, these mutants carried off-target cleavages outside the regions we analyzed, those events should have no effect on the functional phenotypes. Thus, we characterized the phenotypes of the two CRISPR/Cas9 genome-engineered *Chst14^−/−^* mutant mice (+6/−10-bp and −1-bp mutants) and *Chst14* gene trap-KO mice generated in this study.

The birth rate of CRISPR/Cas9 genome-engineered *Chst14* homozygous mice decreased sharply as the generation progressed (birth rate of homozygous mice: F2 generation, 11.9%; F4 generation, 1.7%). The +6/−10-bp and −1-bp mutant *Chst14^−/−^* mice demonstrated delayed growth during early life, with a smaller body mass in both male and female mice compared to that of littermate wild-type mice (*Chst14^+/+^*, *P*<0.05) and heterozygous mice (*Chst14^+/−^*, *P<*0.01) (Fig. 1B,C; Fig. S3A,B). Similar results were obtained for *Chst14* gene trap-KO line (Fig. S4A), as previously reported (Akyüz et al., 2013). We confirmed significantly decreased mRNA levels of *Chst14* in both −1-bp and +6/−10-bp mutant *Chst14^−/−^* mice (Fig. 1D), suggesting that immature mRNA was caused by gene editing and was not detectable in the *Chst14* gene trap-KO mice (Bian et al., 2011).

To investigate the effects of D4ST1 activity on the biosynthesis of DS following *Chst14* editing, we analyzed the amount of CS and DS disaccharides in the skeletal muscle (Fig. 1E; Fig. S5) and urine (Fig. S6). Using high-performance liquid chromatography (HPLC) analysis, DS disaccharides were detected in the skeletal muscle of *Chst14^+/+^* and *Chst14^+/−^* mice, whereas no DS disaccharides were detected in *Chst14^−/−^* mice (–1-bp mutants; versus *Chst14^+/+^* or *Chst14^+/−^*, *P<*0.0001), and a significantly increased level of CS disaccharides was detected (Fig. 1E; Fig. S5). DS disaccharides were also not detected in the urine samples from *Chst14^−/−^* mice (+6/−10-bp mutants) (Fig. S6), similar to the samples from the *Chst14* gene trap-KO mice (Fig. S4B). CS disaccharides were elevated in the urine samples from mutant mice (+6/−10-bp) compared to those from wild-type mice (Fig. S6), which was consistent with the findings from *Chst14* gene trap-KO mice (Fig. S4B). These results suggest that D4ST1 activity is defective in the *Chst14* gene trap-KO and *Chst14* mutants.

### Skin structure and function in *Chst14^−/−^* mutant mice

We did not visually notice significant skin hyperextensibility, bruisability or fragility in *Chst14^−/−^* mice. In order to perform a detailed evaluation of the skin phenotypes in CRISPR/Cas9 genome-engineered *Chst14^−/−^* mutant mice, histological (Fig. 1F; Fig. S7A) and functional (Fig. 1G–I; Fig. S7B) analyses were performed using dorsal skin specimens obtained from aged mice (1 year old). Although the collagen fibrils in the reticular dermis of *Chst14^+/+^* mice formed a tight bundle, the structures of the fiber bundles in the reticular dermis of both −1-bp and +6/−10-bp mutant *Chst14^−/−^* mice were finer, and the boundary between adjacent collagen bundles was unclear (Fig. 1F; Fig. S7A). Similar phenotypes were observed in *Chst14* gene trap-KO mice, as shown in our previous study (Hirose et al., 2021). After immunohistochemical staining of skin specimens from *Chst14^+/+^* and −1-bp mutant *Chst14^+/−^* mice, decorin core protein was observed on collagen fibers in thick bundles with clear boundaries (Fig. 1F). In contrast, we observed decorin core protein in the skin specimens from *Chst14^−/−^* mutant mice on collagen fibers that appeared thin and filamentous without clear boundaries. Moreover, −1-bp mutant *Chst14^−/−^* mice had significantly thinner skin (0.38±0.05 mm) than *Chst14^+/+^* (0.52±0.05 mm; versus *Chst14^−/−^*, *P*<0.0001) and *Chst14^+/−^* (0.51±0.05 mm; versus *Chst14^−/−^*, *P*<0.0001) mutant mice (Fig. 1G).

To examine functional fragility, we measured tensile stress and tensile strain using fresh dermal skin. The −1-bp mutant (Fig. 1H) and +6/−10-bp mutant (Fig. S7B) *Chst14^−/−^* mice showed remarkably weaker tensile stress than the *Chst14*^+/+^ mice (*P*<0.01). In addition, *Chst14^−/−^* mice (−1-bp mutant) showed a significantly lower tensile strain than *Chst14^+/+^* mice (*P*=0.0073) (Fig. 1I). The tensile stress and tensile strain measurements were obtained from the linear slopes of the force–displacement and stress–strain curves, respectively (Fig. S8). The slope of the graph reflects the hardness of the skin, although these curves are non-linear because the skin is a hyperelastic material. The skin of homozygous mutant mice showed greater fragility than that of heterozygous and wild-type mice (Fig. 1F–I). These results suggest that the CRISPR/Cas9 genome-engineered *Chst14^−/−^* mice share common phenotypes, including growth delay and skin fragility, with *Chst14* gene trap-KO mice (Hirose et al., 2021).

### Myopathy findings and reduced muscle function in *Chst14^−/−^* mutant and gene trap-KO mice

**Fig. 2.**
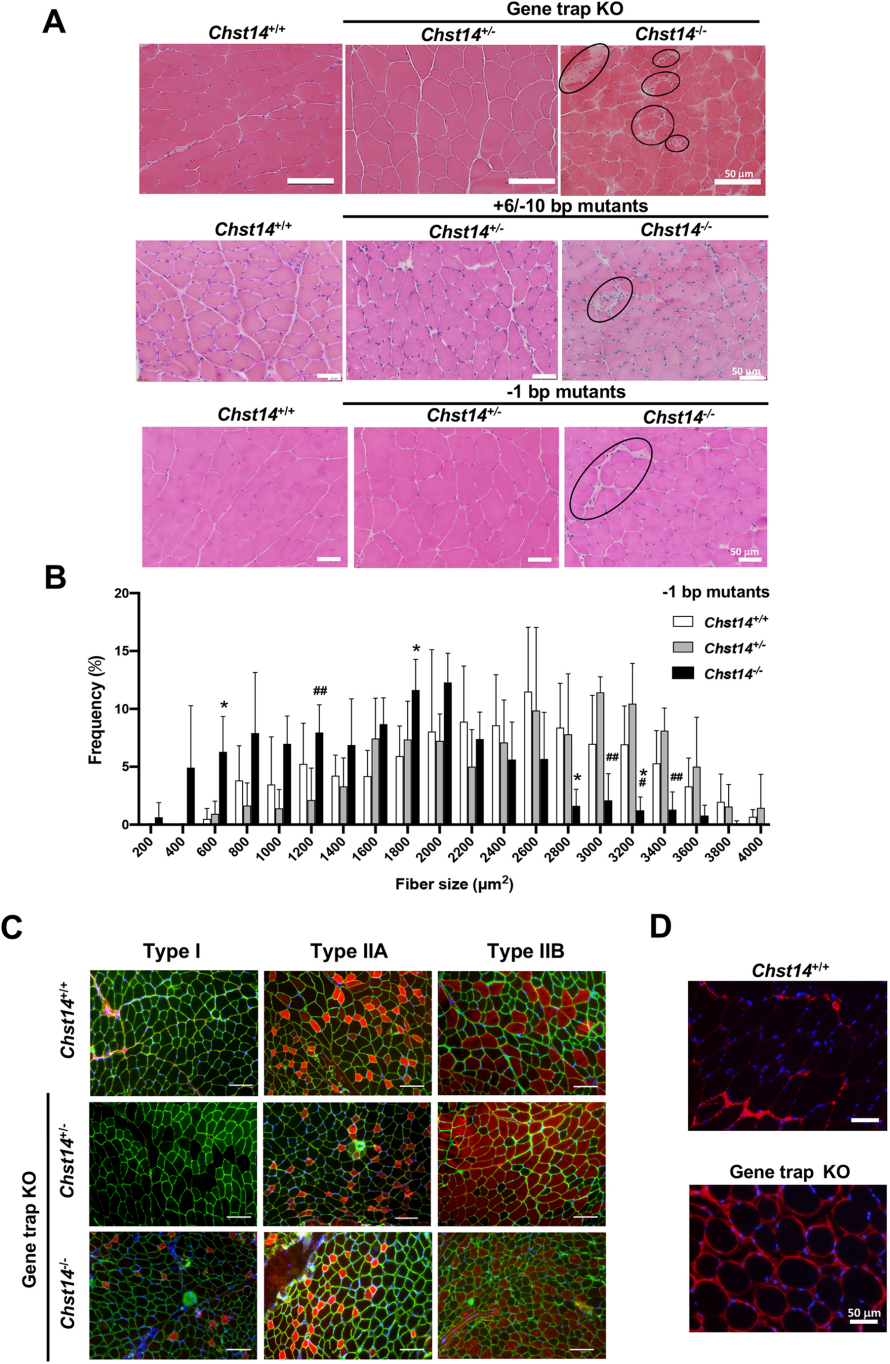
**Pathological phenotypes in the skeletal muscle of *Chst14* mutant and *Chst14* gene trap-KO mice.** (A) H&E staining of the tibialis anterior muscle from *Chst14^+/+^*, *Chst14^+/−^* and *Chst14^−/−^* mice (top row, gene trap-KO; middle row, +6/−10-bp mutants; bottom row, −1-bp mutants). Circles show the nucleic accumulation and spread muscle fiber stroma. Scale bars: 50 μm. (B) Frequency distribution of myofiber area (µm^2^) counted from H&E-staining images of the tibialis anterior muscle from *Chst14^+/+^* (2 males and 4 females), −1-bp mutant *Chst14^+/−^* (2 males and 2 females) and *Chst14^−/−^* (4 females) mice. Area values show total fibers (numbers/5.5 mm^2^) and distribution comparisons. Data are presented as mean±s.d. Statistical differences compared to *Chst14^+/+^* (*P<0.05) and *Chst14^+/−^* (^#^*P*<0.05 and ^##^*P*<0.01) mice were evaluated using multiple comparisons. (C) Immunofluorescence staining of the tibialis anterior muscle from *Chst14^+/+^* mice and *Chst14^+/−^* and *Chst14^−/−^* gene trap-KO mice performed using anti-MHC for detection of type I (slow muscle), IIA and IIB muscle fibers (fast muscle). Red signals indicate stained areas. Scale bars: 100 μm. (D) Immunofluorescence staining of the tibialis anterior muscle of *Chst14^+/+^* and *Chst14^−/−^* gene trap-KO mice; red signals indicate decorin, blue signals indicate nuclei (4,6-diamidino-2-phenylindole staining). Scale bars: 50 μm.

We next performed a systemic investigation of muscle phenotypes in mcEDS using *Chst14* gene trap-KO mice and CRISPR/Cas9 genome-engineered *Chst14^−/−^* mutant mice. First, we were able to visually confirm that the muscle mass in the forelimb skeletal muscle of +6/−10-bp mutant *Chst14^−/−^* mice, as well as that of gene trap-KO mice, was lower than that in the corresponding muscle of age-matched *Chst14^+/+^* and *Chst14^+/−^* mice (Fig. S9A,B). Therefore, we analyzed muscle histopathology to examine myopathy caused by D4ST1 deficiency. A cross-section of the tibialis anterior (TA) muscle from *Chst14* gene trap-KO mice and *Chst14^−/−^* mutant mice (+6/−10-bp and −1-bp mutants) revealed spreading of the muscle interstitium, and Hematoxylin and Eosin (H&E) staining revealed cell infiltration (Fig. 2A). In addition, we observed an increased frequency of smaller-diameter fibers in the −1-bp mutant *Chst14^−/−^* mice (Fig. 2B), +6/−10-bp mutant *Chst14^−/−^* mice (Fig. S10A) and *Chst14* gene trap-KO mice (Fig. S10B) than in the respective *Chst14^+/+^* and *Chst14^+/−^* mutant mice. Immunofluorescence of myosin heavy chain (MHC) isoforms showed that the muscle fibers in the TA muscle of *Chst14^+/+^* and heterozygous *Chst14* KO mice were primarily type IIA/B-positive (fast muscle) fibers, with only 1% type I-positive (slow muscle) fibers (Fig. 2C). In contrast, *Chst14* gene trap-KO mice showed a larger number of MHC type I-positive fibers than *Chst14^+/+^* and *Chst14^+/−^* mice (Fig. 2C), suggesting that the muscle fiber type might be affected by D4ST1 deficiency. Furthermore, to determine the location of decorin in the skeletal muscle of *Chst14* gene trap-KO mice, immunofluorescence analysis was performed on the TA muscle sections (Fig. 2D). The *Chst14^+/+^* section revealed positive immunostaining for decorin in the perimysium; in the endomysia, it was poorly detected. In contrast, decorin reactivity in the muscle of *Chst14* KO mice was upregulated in the perimysium around packages of muscle fibers and was also augmented around individual muscle fibers in the endomysium. We did not observe a significant difference in the serum levels of CK, which is known as a marker of muscle damage, in 1-year-old *Chst14^+/+^* (20±9.0 ng/ml) and *Chst14^−/−^* gene trap-KO mice (29.9±16.9 ng/ml, *n*=3, *P*=0.498).

**Fig. 3.**
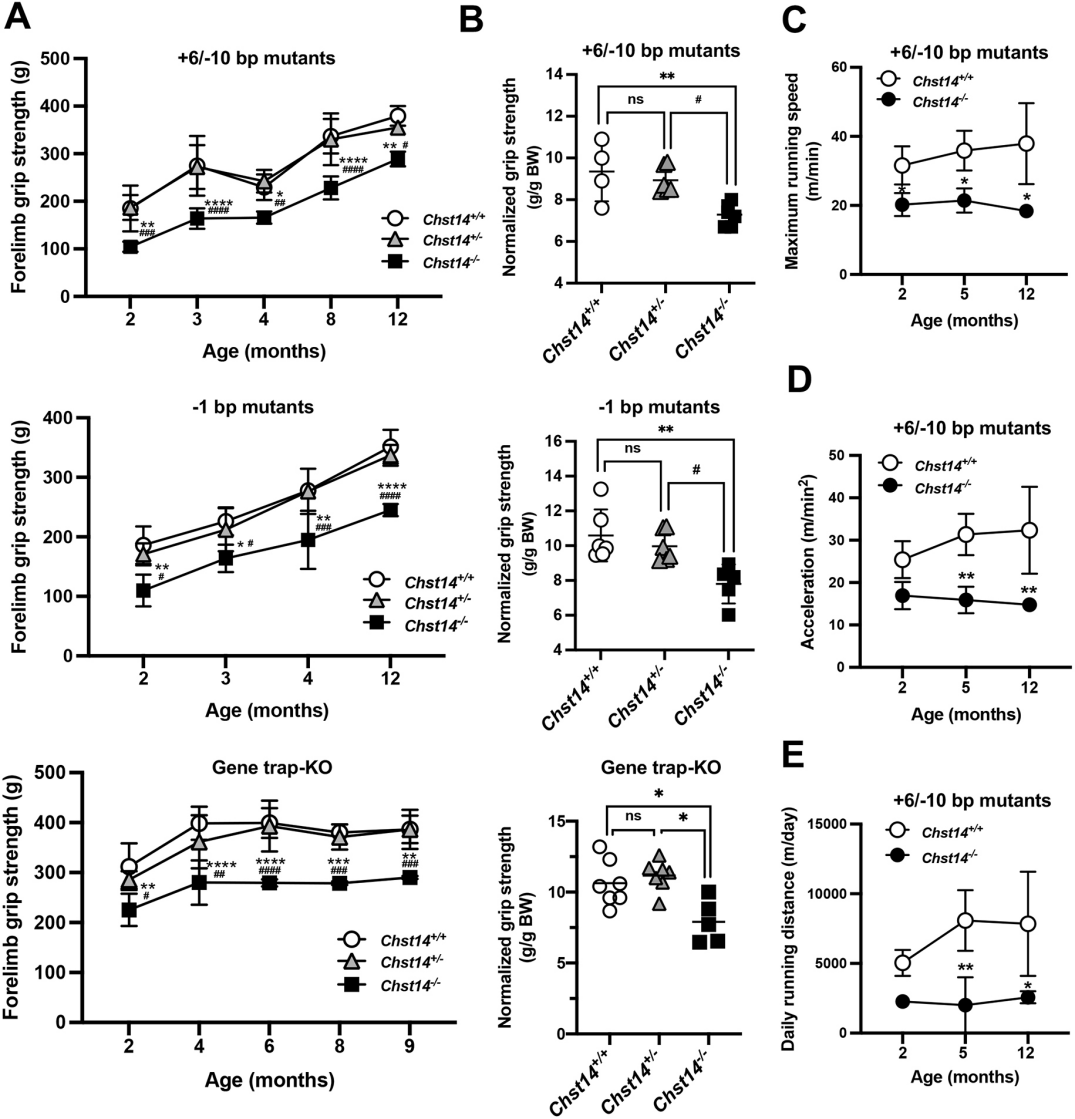
**Decreased grip strength and voluntary activity of *Chst14*-mutant and -KO mice.** (A) Grip strength data (***g***) for 2- to 12-month-old *Chst14^+/+^* (top, 2 males and 2 females; middle, 1 male and 3 females; bottom, 3 males and 2 females), *Chst14^+/−^* (top, +6/−10-bp mutant, 5 males and 1 female; middle, −1-bp mutant, 2 males and 5 females; bottom, gene trap-KO, 6 males and 6 females) and *Chst14^−/−^* (top, +6/−10-bp mutant, 2 males and 3 females; middle, −1-bp mutant, 2 males and 1 female) mutant mice and 2- to 9-month-old *Chst14* gene trap-KO mice (bottom, 2 males and 1 female). (B) Normalized grip strength [***g***/g body weight (BW)] measured in 2-month-old *Chst14^+/+^* (top, 2 males and 2 females; middle, 4 males and 2 females), *Chst14^+/−^* (top, +6/−10-bp mutant, 5 males and 1 female; middle, −1-bp mutant, 1 male and 5 females) and *Chst14^−/−^* (top, +6/−10-bp mutants, 2 males and 3 females; middle, −1-bp mutants, 4 males and 1 female) mutant mice, and in 1-year-old *Chst14^+/+^* (bottom, 3 males and 4 females), *Chst14^+/−^* (bottom, 5 males and 2 females) and *Chst14^−/−^* gene trap-KO (bottom, 1 male and 4 females) mice. (C–E) *Chst14^+/+^*and +6/−10-bp mutant mice (2, 5 and 12 months old) analyzed using voluntary running activity in the wheel cage that yielded the maximum running speed (m/min) (C), acceleration (D) and daily running distance (E) for *Chst14^+/+^* (1 male and 2 females), +6/−10-bp mutant *Chst14^+/−^* (2 males and 1 female) and *Chst14^−/−^* (3 females) mice. Data are presented as mean±s.d. Statistical differences, compared to *Chst14^+/+^* (*P<0.05, **P<0.01, ***P<0.001 and ****P<0.0001) and *Chst14^+/−^* (^#^*P*<0.05, ^##^*P*<0.01, ^###^*P*<0.001 and ^####^*P*<0.0001), were evaluated using one- or two-way ANOVA. ns, not significant.

We next investigated the pathological effects of non-production of DS on muscle histology and function using *Chst14^−/−^* mutant mice. To assess whether histopathological findings were associated with motor function, we performed an analysis of grip strength (Fig. 3A,B), voluntary activity and running speed (Fig. 3C–E). By monitoring grip strength data, we observed that the *Chst14^−/−^* mutant mice (+6/−10-bp and −1-bp mutants) showed significantly lower strength during the experimental period than age- and sex-matched *Chst14^+/+^* mice (*P*<0.05) and *Chst14^+/−^* mice (*P*<0.05) (Fig. 3A,C; Fig. S3C). In addition, we observed a reduction in body weight-normalized grip strength in 2-month-old +6/−10-bp mutant *Chst14^−/−^* mice (versus *Chst14^+/+^*, *P*=0.007; versus *Chst14^+/−^*, *P*=0.014) and −1-bp mutant *Chst14^−/−^* mice (versus *Chst14^+/+^*, *P*=0.005; versus *Chst14^+/−^*, *P*=0.025) (Fig. 3B). Similar results were also observed in the *Chst14* gene trap-KO mice (Fig. 3A,B). These results demonstrate that reduced grip strength is a common characteristic of *Chst14* gene trap-KO and *Chst14^−/−^* mutant mice, indicating reduced muscle function caused by the loss of D4ST1.

To better understand the effect of motor function on *Chst14* gene trap-KO and *Chst14^−/−^* mutant mice, voluntary activity was measured in young and adult mice (Fig. 3C–E; Fig. S11). The results indicate that the maximum running speed of *Chst14* gene trap-KO mice (47.7±1.0 m/min) was lower than that of *Chst14^+/+^* mice (69.0±8.8 m/min, *P*=0.029) (Fig. S11A,B). Similar to the gene trap-KO mice, the maximum running speed of *Chst14^−/−^* mutant mice (2- to 12-month-old +6/−10-bp mutant mice) was also lower than that of *Chst14^+/+^* mice (*P*<0.05) (Fig. 3C), which was supported by the acceleration data (5- to 12-month-old *Chst14^−/−^* mice, *P*<0.05) (Fig. 3D). Comparing the time spent in the wheel cage, the daily running distance of +6/−10-bp mutant *Chst14^−/−^* mice was significantly lower than that of *Chst14^+/+^* mice (5 months old, *P*<0.001) (Fig. 3E). Similar results were obtained with 1-year-old −1-bp mutant *Chst14^−/−^* mice (Fig. S11C,D).

### Thoracic kyphosis in *Chst14^−/−^* mutant mice

**Fig. 4.**
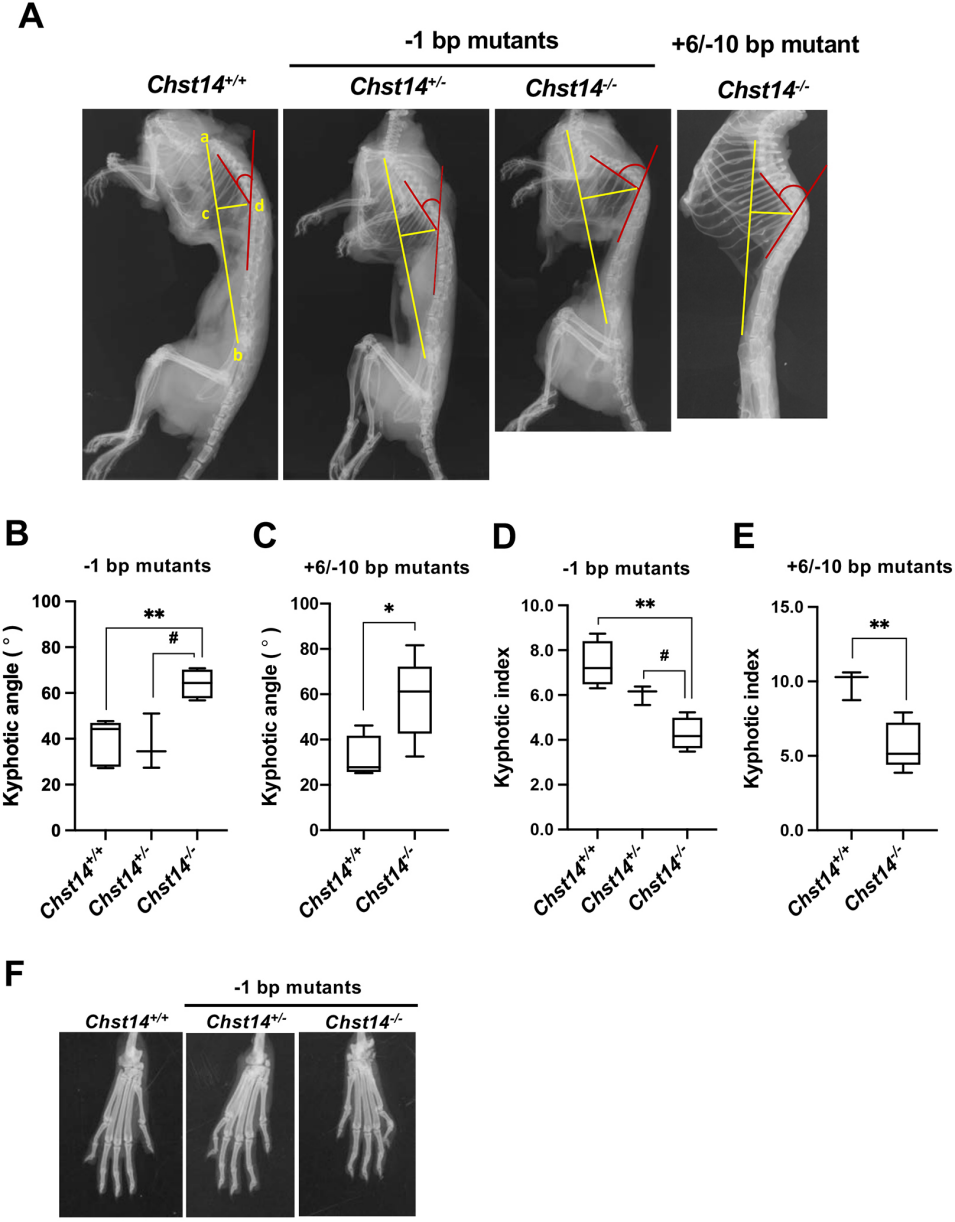
**Thoracic kyphosis in *Chst14*-mutant mice.** (A) Representative lateral radiographs for 1-year-old female *Chst14^+/+^*, −1-bp mutant *Chst14^+/−^* and *Chst14^−/−^* mutant (−1-bp and +6/−10-bp mutants) mice. (B,C) Kyphotic angle calculated by measuring the angle indicated in red on the radiographs for *Chst14^+/+^* (2 males and 3 females), −1-bp mutant *Chst14^+/−^* (2 males and 1 female) and *Chst14^−/−^* (1 male and 3 females) mice (B), and *Chst14^+/+^* (2 males and 2 females) and +6/−10-bp mutant *Chst14^−/−^* (1 male and 4 females) mice (C). (D,E) Kyphotic index calculated using distances a–b and c–d (yellow lines a−b/c−d) in *Chst14^+/+^* (2 males and 2 females), −1-bp mutant *Chst14^+/−^* (2 males and 1 female) and *Chst14^−/−^* (1 male and 3 females) mice (D), and *Chst14^+/+^* (1 male and 2 females) and +6/−10 -bp mutant *Chst14^−/−^* (1 male and 4 females) mice (E). (F) Radiographs of forefoot fingers of 1-year-old female *Chst14^+/+^* and −1-bp mutant (*Chst14^+/−^* and *Chst14^−/−^*) mice. Data are presented as mean±s.d. Statistical differences, compared to *Chst14^+/+^* (*P<0.05 and **P<0.01) and *Chst14^+/−^* (^#^*P*<0.05), were evaluated using one-way ANOVA or unpaired two-tailed Student's *t-*test.

Marked thoracic kyphosis was observed over time, and the phenotype became prominent at 1 year of age. Therefore, we analyzed thoracic kyphosis using lateral spinal radiographs in aged *Chst14^−/−^* mice (−1-bp and +6/−10-bp mutant mice), compared to *Chst14^+/+^* and *Chst14^+/−^* mutant mice (Fig. 4A). To further quantify the severity of kyphotic deformity in *Chst14^−/−^* mutant mice, we analyzed the kyphotic angle (Fig. 4B,C) and kyphotic index (distance c–d/a–b) (Fig. 4D,E). These data were measured in anterior–posterior radiographs to objectively identify scoliosis and to determine the severity of the lateral curvature of the spine. The −1-bp mutant *Chst14^−/−^* mice showed significantly lower thoracic angles than *Chst14^+/+^* mice (*P*=0.009) and *Chst14^+/−^* mice (*P*=0.014) (Fig. 4B), equal to those of +6/−10-bp mutant *Chst14^−/−^* mice (versus *Chst14^+/+^*, *P*=0.033) (Fig. 4C). *Chst14^−/−^* mutant mice (−1-bp mutant) also showed a large decrease in the kyphotic index (versus *Chst14^+/+^*, *P*=0.0015; versus *Chst14^+/−^*, *P*=0.047) (Fig. 4D), as did the +6/−10-bp mutant *Chst14^−/−^* mice (versus *Chst14^+/+^*, *P*=0.033) (Fig. 4E). Morphological changes in the spinal curvature were also visually observed in the gene trap-KO mice (Fig. S9C). Digital contractures were not observed using X-ray imaging of the forefoot fingers of the −1-bp mutant (Fig. 4F) and +6/−10-bp mutant (Fig. S9D) *Chst14^−/−^* mice, and gene trap *Chst14^−/−^* KO mice (Fig. S9E). These data demonstrate that thoracic kyphosis was a characteristic phenotype in *Chst14^−/−^* mutant mice, although X-ray images did not show any significant abnormality in joint contracture.

## DISCUSSION

In the present study, we successfully developed a *Chst14* mutant mouse using CRISPR/Cas9-mediated gene engineering and *Chst14* gene trap-KO mice with a united genetic background (C57BL/6J). There are no null mutants in humans. However, it was unclear whether the *Chst14* gene-edited mice we created would lose D4ST1 function. To investigate whether the mutant mice share an mcEDS phenotype, a comprehensive analysis was performed comparing the two generated *Chst14* mutants and the *Chst14* gene trap-KO mice. In this study, we showed evidence of the loss of function of D4ST1 activity and the pathological phenotypes caused by *Chst14* mutation by comparing *Chst14* mutant mice and gene trap-KO mice. These two *Chst14^−/−^* mutant mice demonstrated a common pathological phenotype, sharing typical features of the mcEDS phenotype, including loss of DS disaccharides, growth delay, skin fragility, myopathy, reduced muscle function and thoracic kyphosis. This is the first study to characterize myopathy caused by the loss of D4ST1 activity in a mouse model.

The characteristics of motor function in the *Chst14* gene trap-KO mice were comprehensively assessed. However, only a limited number of adult mice were generated as a consequence of perinatal lethality in most homozygous gene trap-KO mice. This issue was explained in our previous report, in which we demonstrated that placental dysplasia has profound effects on the production of homozygous mice (Yoshizawa et al., 2018). Perinatal lethality has rarely been reported in patients with mcEDS-*CHST14* (Dündar et al., 2009), while perinatal lethality is common in mouse models, presumably due to placental factors (Yoshizawa et al., 2018). This phenotype may be rodent specific and may also be affected by genetic background. It is better to produce *Chst14^−/−^* mutant homozygous mice from the F1 and F2 generations than from continuous generation, and they can be useful compared to *Chst14^−/−^* gene trap-KO mice. In the current study, we propose an efficient method to generate *Chst14^−/−^* mutant mice created by CRISPR/Cas9 genome engineering as an mcEDS model mouse.

We confirmed that *Chst14* mRNA was largely decreased and no DS disaccharides were present in the *Chst14^−/−^* mutant mice. These characteristics appeared to be due to complete inactivation of the D4ST1 enzyme in the *Chst14^−/−^* mutant mice. Similar observations were made for urine samples from patients with mcEDS-*CHST14* (Mizumoto et al., 2017). Loss of D4ST1 (and thus DS chains) also caused structural fragility of the skin, as a consequence of altered collagen fibril morphology. As reported in previous studies with *Chst14* gene trap-KO mice, the skin of *Chst14^−/−^* mutant mice demonstrated low resistance to tensile stress, suggesting that skin fragility was a common phenotype in all *Chst14^−/−^* mutants (Fig. 1; Fig. S7).

We also identified several myopathy phenotypes in *Chst14^−/−^* mice, including a predominance of small muscle fiber size and type I muscle fibers, both of which are characteristic phenotypes found in patients with mcEDS (Dundar et al., 1997; Kosho et al., 2010; Voermans et al., 2012). These phenotypes probably contributed to the gross motor developmental delay in mcEDS. CK levels were mildly elevated in patients (CK 277U/L; N<160 U/l) (Voermans et al., 2012), but *Chst14^−/−^* mutant mice did not show an increase in CK levels. The D4ST1 expression pattern in the skeletal muscle of mcEDS and its biological effects have not previously been reported. In the present study, we report a drastic reduction in DS disaccharides in the TA muscle of *Chst14^−/−^* mutant mice and a concomitant increase in the amount of CS (Fig. 1E). Consequently, the decorin GAG side chains in this mcEDS model comprised only CS, not DS, disaccharides. It was previously reported that collagen bundles bound by CS chains are more fragile than the bundles in normal chains (which are bound by CS/DS chains), because CS/DS hybrid chains are more flexible than CS chains (Miyake et al., 2010). These observations suggest that the structural fragility of skeletal muscle is affected by connective tissue fragility, similar to skin structural fragility.

An altered localization of decorin was observed in the muscle of *Chst14* mutant mice (Fig. 2D). Decorin is a small CS/DS proteoglycan belonging to a family of structurally related proteoglycans, which are the main constituents of the extracellular matrix (ECM). Decorin is well known to function in collagen fibrillogenesis and tissue homeostasis (Reed and Iozzo, 2002). Altered localization of decorin with CS may be caused by connective tissue fragility in the tissue associated with ECM functional changes. The presence of ECM is essential for normal myogenesis, which includes interactions between myoblasts and their environment (Osses and Brandan, 2002). In the case of dystrophic skeletal muscle, biosynthesis and accumulation of decorin around individual muscle fibers were enhanced in the endomysium and exomysium (Cáceres et al., 2000). In fact, expression of decorin mRNA was confirmed in the connective tissue cells (i.e. mesenchymal cells and satellite cells), suggesting that decorin plays an important role in organizing the fibrillar network of the ECM (Cáceres et al., 2000; Fadic et al., 2006). Various proteoglycans, either at the plasma membrane or in the ECM, have been reported to play a role in the differentiation process by regulating growth factor activity (Villena and Brandan, 2004). For example, the hepatocyte growth factor-dependent migratory process requires the presence of proteoglycans/sulfated GAGs on the myoblast surface, and hepatocyte growth factor-dependent myoblast migration is increased largely by DS. Thus, DS is considered an enhancer of growth factor-dependent proliferation of satellite cells and migration during skeletal muscle formation (Villena and Brandan, 2004). Hence, DS depletion in the skeletal muscle in *Chst14*^−/−^ as well as in mcEDS may induce abnormal collagen bundle formation associated with decorin GAG abnormality, resulting in several histological phenotypes including increased fiber size variation, fiber-type predominance and spread intermedium. Considering these results, the myopathy phenotype in *Chst14^−/−^* and mcEDS may be caused by connective tissue fragility in the skeletal muscle associated with ECM functional changes, including changes in decorin localization and pathological activation.

In patients with mcEDS, muscle hypoplasia and changes in dynamometer muscle force have been reported, resulting in lower grip strength in these patients than in healthy individuals (Voermans et al., 2012). This myopathy probably contributes to the gross motor developmental delay in this type of EDS. Muscle weakness in *Chst14^−/−^* mice was also associated with reduced grip strength and locomotor activity (Fig. 3). Although locomotor activity and motor function both increased with growth in wild-type mice, they did not increase in *Chst14* gene trap-KO and mutant mice (Fig. 3; Fig. S11). The reduced running distance and maximum running speed in *Chst14^−/−^* mutant mice reflect a decrease in both continuous motor ability and voluntary motor function (Fig. 3).

Although low levels of grip strength could be related to myopathy, it is also necessary to consider the effect of digital contractures. In this study, digital contractures were not observed in the forefoot fingers of *Chst14^−/−^* mutant mice (Fig. 4F). Further research is needed to explain the digital contracture phenotypes in *Chst14^−/−^* mutant mice. Muscle weakness generally enhances kyphosis severity. Indeed, patients with mcEDS are reported to present with myopathy accompanied by muscle hypoplasia and muscle weakness, similar to other EDS types (Voermans et al., 2012). EDS caused by mutations in *FKBP14* also shows myopathy and progressive kyphoscoliosis (Baumann et al., 2012). Here, we demonstrate that kyphosis, which is associated with myopathy in our mouse models, mirrors the mcEDS phenotype (Mendoza-Londono et al., 2012; Uehara et al., 2018, 2020).

Using *Chst14^−/−^* mutant mice developed by CRISPR/Cas9 genome engineering, we were able to comprehensively investigate the pathological mechanisms associated with the loss of D4ST1 and DS chains. To achieve continuous maintenance of an mcEDS model, further studies are planned, including backcrossing of the C57BL/6J strain to a BALB/c strain in which the birth rate of *Chst14* KO mice was improved, suggesting that the genetic background influences the birth rate of *Chst14*^−/−^ mice (Shimada et al., 2020). Furthermore, conditional KO will also be attempted using the Cre-loxP system. These mcEDS models should facilitate future research on the pathological analysis of mcEDS and on potential therapeutic approaches, such as enzyme replacement therapy.

## MATERIALS AND METHODS

### Animals

All experimental procedures were approved by the Experimental Animal Care and Use Committee at the National Center of Neurology and Psychiatry (NCNP) and Nippon Medical School. All animals were maintained in accordance with the standard protocol for animal care at the NCNP and Nippon Medical School, and used in accordance with local, national and international regulations and guidelines. Gene trap-*Chst14* KO mice were obtained from the Mutant Mouse Regional Resource Center (https://www.mmrrc.org) (Bian et al., 2011; Tang et al., 2010) and bred for more than 12 generations. The mice were housed in a microisolator (Shin Toyo Seisakusho, Kawaguchi, Japan) at 23±2°C with constant humidity and a 12 h light/dark cycle. The animals had free access to tap water and standard mouse chow (Funabashi Farm, Funabashi, Japan). *Chst14* gene trap-KO mice were inbred for eight generations (Trans Genic Inc., Fukuoka, Japan) as C57BL/6J and 129S2/SvImJ mixed background. C57BL/6J, 129S1/SvImJ and C57BL/6J/C3F1 mice were purchased from The Jackson Laboratory (Bar Harbor, ME, USA), Nihon CLEA (Tokyo, Japan) and Japan SLC (Shizuoka, Japan), respectively. Age-matched littermate mice were used in all the experiments.

### Generation of *Chst14* gene trap-KO mouse

The lines of *Chst14* KO*/*C57BL/6J mice were established by breeding individuals with high replacement rates for SSLP markers (Table S1). To discriminate between the 129S2/SvImJ and C57BL/6J mouse, 58 strain-specific SSLP markers were used; DNA from mouse tail was amplified by PCR using SSLP-specific primers and Ex Taq DNA polymerase or Tks Gflex DNA polymerase (Takara Bio Inc., Shiga, Japan) according to a previously reported method (Lamacchia et al., 2007). PCR products were resolved by agarose gel electrophoresis. Heterozygous mice with the highest replacement rates for 129S2/SvImJ mice markers into C57BL/6J mice markers were selected for mating to obtain the next generation. Homozygous *Chst14* KO mice were maintained as an mcEDS model.

### Generation of the *Chst14* mutant mouse by CRISPR/Cas9 genome editing

To perturb the *Chst14* coding sequence by introducing DSBs via the CRISPR/Cas9 system, the region downstream from the translational start site ATG in the 1st exon was targeted (Fig. 1A). Six sgRNAs (#1, #3, #5, #6, #7 and #8; Fig. S1A) were designed using the web-based CRISPR design tool, CRISPOR (http://crispor.tefor.net/) (Concordet and Haeussler, 2018). The sgRNA sequences were cloned into the *Bbs*I site of the pX330 vector (Addgene plasmid #42230). To evaluate the DNA-cleavage activities of these sgRNAs, the pCAG-EGxxFP system (Mashiko et al., 2013) was employed. The *Chst14* genomic region (600 bp) spanning the sgRNA target sequence was cloned between the overlapped 5′ and 3′ EGFP fragments within the pCAG-EGxxFP vector (Addgene plasmid #50716), which could be used for sgRNA validation by DSB-mediated EGFP reconstitution. This substrate plasmid and the Cas9/sgRNA-expressing pX330 were co-transfected into HEK293EB cells using Lipofectamine^®^ 2000 Reagent (Thermo Fisher Scientific, Waltham, MA, USA). pCAG-EGxxFP-Cetn1 (Addgene plasmid #50717) and pX330-Cetn1/sgRNA1 (Addgene plasmid #50718) were used as positive controls, whereas empty pX330 served as the negative control. The efficiency of homology-dependent repair was validated by observing EGFP fluorescence 48 h after transfection using an IX81 microscope (Olympus, Tokyo, Japan).

Fertilized eggs were collected from C57BL/6J/C3F1 mice (Japan SLC). The circular pX330 vectors containing sgRNA #6 or #7 were directly injected into the pronuclei of the one-cell embryos according to previously reported protocols (Mashiko et al., 2013), and the injected eggs were transferred into the oviduct of pseudo-pregnant ICR females to raise KO founders. Newborns were screened by PCR amplification of the sgRNA target region. The PCR products were analyzed by Sanger sequencing to evaluate various genome-editing events (Table S3). One knock-in allele from the sgRNA #6 injection and another knock-in allele from the sgRNA #7 injection were selected to establish the KO founders (Fig. 1A; Fig. S1A), which were crossed with wild-type mice to obtain the F1 generations. The F1 heterozygous mice were further intercrossed to maintain heterozygous and homozygous mutant mice.

To exclude the unintended side effects from possible off-target cleavages, the genomic DNA extracted from the founder mice was analyzed by Sanger sequencing. CRISPOR (Concordet and Haeussler, 2018) was employed to predict the off-target candidate loci, and five loci for each sgRNA (#6 and #7) listed in Table S4A were amplified by PCR from the founders’ genomic DNA and then analyzed by sequencing. The primers used are listed in Table S4B.

### Mouse genotyping

Genomic DNA was extracted for genotyping of the mouse tails. The gene trap-KO or −1-bp and +6/−10-bp mutant alleles of *Chst14* were confirmed by *Chst14*-amplification-resistant mutation system PCR using Ex Taq DNA polymerase (Takara Bio Inc.) with a universal forward primer, 5′-CCACTGGACCTGTTTAAGCAGG-3′, and a reverse primer for wild-type recognition, 5′-GTGTGCAATTCTACTCCAGACT-3′, or a reverse primer for null mutant recognition, 5′-GCAGCGCATCGCCTTCTATC-3′. PCR conditions were as follows: 95°C for 2 min; 32 cycles of 95°C for 10 s, 60°C for 30 s, and 72°C for 45 s; and a final extension at 72°C for 2 min. To compare the genotyping results, the PCR products were subjected to agarose gel electrophoresis. To detect *Chst14* mutant mice, PCR products were amplified using the forward primer, 5′-CCACTGGACCTGTTTAAGCAGG-3′, and reverse primer, 5′-GTGTGCAATTCTACTCCAGACT-3′. PCR conditions were as follows: 95°C for 2 min; 30 cycles of 95°C for 30 s, 66.8°C for 30 s, and 72°C for 30 s; and a final extension at 72°C for 5 min. Genotyping to detect mutated alleles was performed using sequence analysis.

#### Quantitative polymerase chain reaction (qPCR)

Total RNA was isolated from tissue samples disrupted in a Multi-Beads Shocker (Yasui Kikai, Osaka, Japan) using an RNeasy Micro Kit (Qiagen). First-strand cDNA was synthesized using a Super Script III First Strand Synthesis System (Thermo Fisher Scientific). The qPCR assay was performed using 1 µg cDNA. The primers used for *Chst14* were as follows: forward, 5′-GCATTACCACTTGTGCAATGTTCCA-3′, and reverse, 5′-GGTGACAGGCTTCCTTGGTGACA-3′. As an internal control, a primer set for the housekeeping gene, glyceraldehyde-3-phosphate dehydrogenase (*Gapdh*) was used as follows: mouse, forward, 5′-GATGACATCAAGAAGGTGGTGA-3′, and reverse, 5′-TGCTGTAGCCGTATTCATTGTC-3′. qPCR was performed using SYBR^®^ Premix Ex Taq™ II (Perfect Real Time, Takara Bio Inc.). SYBR green detection of PCR products was conducted in real time using a MyiQ single-color detection system (Bio-Rad, Hercules, CA, USA).

### Histopathology and immunohistochemistry

Histological analysis was performed using four mice in each mouse group. Dorsal skin samples obtained from the back of each mouse were fixed in 20% neutral buffered formalin solution. Paraffin sections (5–6 µm thickness) were prepared and fixed with 10% formaldehyde or 4% paraformaldehyde, and H&E staining and immunostaining for decorin were performed. The TA muscle taken from 1-year-old mice was immediately frozen in liquid nitrogen-cooled isopentane. Transverse cryosections (10 μm thickness) were prepared from the frozen muscle tissues, stained with H&E using standard procedures and immunostained for decorin. After microwave treatment with citrate buffer (pH 6.0) or treatment with 0.1% proteinase K for antigen activation, endogenous peroxidase was removed using hydrogen peroxide-containing methanol, and rabbit serum was used to block non-specific reactions. Immunohistochemistry was performed using a 1:200 dilution of biotinylated anti-mouse decorin antibody (polyclonal goat IgG, Fujifilm, Osaka, Japan) as the primary antibody and peroxidase-labeled streptavidin (Dako, Glostrup, Denmark, or Nichirei, Tokyo, Japan) as the secondary antibody, and then developed using 3,3-diaminobenzidine tetrahydrochloride hydrate solution (Nichirei). All samples were visualized using a light microscope (AX80 or CKX41, Olympus). Muscle cryosections fixed with 1% paraformaldehyde were treated with a 1:100 dilution of anti-mouse decorin antibody (mouse monoclonal IgG1 clone, Fujifilm), or anti-mouse MHC type I (1:60), IIA (1:10) or IIB (1:5) monoclonal antibodies (Abcam, Cambridge, UK), followed by a 1:500 dilution of Alexa Fluor 568-conjugated anti-mouse IgG2b, IgG1 or IgM antibodies, or IgG antibody conjugated with Alexa Fluor 488 (Thermo Fisher Scientific) as the secondary antibody, in which the Alexa Fluor secondary antibodies were used for immunohistochemistry. The immunostained sections were mounted in Vectashield with 4, 6-diamidino-2-phenylindole (Vector Laboratories). Immunofluorescence and H&E staining were visualized using an IX81 fluorescence microscope (Olympus). Quantitative analysis of myofibers in the H&E images was performed using CellSence software (Olympus). Three animals from each group were selected for the determination of muscle fiber size, analyzing three sections from each mouse (total 122–196 fibers/mouse). For quantification analysis of myofiber size distribution using immunohistochemistry, muscle sections were treated with a 1:500 dilution of anti-laminin monoclonal antibody (Alexis Biochemical, Southern Ontario, Canada) and a 1:500 dilution of Alexa Fluor 488-conjugated anti-rat IgG antibody (Thermo Fisher Scientific). Myofiber size was evaluated from the laminin fluorescence signal using a fluorescence microscope BZ-9000 (Keyence, Osaka, Japan).

### Body weight and grip strength

The body weight and grip strength of age- and sex-matched *Chst14^+/+^*, *Chst14^+/−^* and *Chst14^−/−^* mutant mice were measured during the experiments. Forelimb grip strength was measured using a grip strength meter (MK-380 M; Muromachi Kikai, Tokyo, Japan) as previously described (Hyzewicz et al., 2015), according to the protocol (IMPC_GRS_001; https://www.mousephenotype.org/impress/ProcedureInfo?action=list&procID=1130) proposed by the International Mouse Phenotyping Consortium. Five measurements, a continuous measurement at 5 s intervals, were performed for each mouse. The average tension force (***g***) was calculated for each group of mice by averaging the three highest measured values out of the five consecutive measured values.

### Analysis of locomotor activity

Physiological activity in the age- and sex-matched mice in each group was analyzed using a computerized wheel system (dual activity monitor system, Shinfactory, Fukuoka, Japan) in each individual cage, and by counting the number of wheel revolutions during every 5 min interval using Actimo-data II software according to our previous report (Nitahara-Kasahara et al., 2021). The average daily running distance was calculated for the mice over 5 days and nights (12 h light/dark cycles). The wheel was continuously used during observation, and the data from the first day were deleted as the habituation period to reduce the influence of preference. The running speed was calculated by converting the mileage for 5 min into mileage for every minute. The maximum speed was calculated by averaging the fastest speed for 5 days. Acceleration was automatically calculated by correcting the fastest speed of the day by time (min) to reach it. The maximum acceleration was calculated by averaging the highest values for each of the 5 days.

### Tensile strength

The skin tensile strength was assessed as previously described (Hirose et al., 2020). Briefly, two pieces of fresh dorsal skin from each group of mice at 1 year old were cut to a size of 10×40 mm, and their thickness was measured with an electronic caliper. The maximum tensile strength was measured using an EZ-S500 N tensile tester (Shimadzu, Kyoto, Japan). The 20 mm length of the front panel was fixed to the jig, and the resistance strength was measured when the tissue was pulled at a speed of 20 m/min. Because tensile stress was corrected by the area of the skin, it was possible to compare the strength without using the difference in the thickness of the skin. The tensile strain indicates the extension rate of the skin piece during the measurement. The tensile stress and tensile strain (the two key measures) were obtained from the linear slopes of the force–displacement and stress–strain curves, respectively. A large inclination indicates that the skin is hard, whereas a small inclination indicates that the skin is soft.

### Quantitative analysis of CS and DS disaccharides

The disaccharide compositions of the CS and DS moieties of CS/DS hybrid chains in the skin, urine and skeletal muscle of age-matched (1-year-old) mice were assessed as described previously (Mizumoto and Sugahara, 2012). Briefly, the GAG fraction was crudely purified from tissue and then digested with a mixture of chondroitinase AC-I and AC-II, or chondroitinase B. Each digest was labeled with a fluorophore, 2-aminobenzamide, and then analyzed by anion-exchange HPLC on a PA-G silica column (4.6×150 mm; YMC Co., Kyoto, Japan). Identification and quantification of the resulting disaccharides were achieved by comparison with the elution positions of the CS- or DS-derived authentic unsaturated disaccharides. The amounts of disaccharides in each sample were calculated by comparing the peak area and the peak area of standard unsaturated disaccharides. CS and DS disaccharides in the muscle and urine were normalized using protein and creatinine levels, respectively.

### Radiography

Whole spine lateral, forelimb and hindlimb radiographs were recorded on a µFX-1000 film (Fujifilm). Imaging conditions were as follows: tube voltage, 25 kV; tube current, 100 µA; exposure time, 30 s (Nakamura-Takahashi et al., 2020). The forelimbs, forefoot fingers and whole spine lateral at 1 year of age were X-ray irradiated and imaged with a Typhoon FLA-7000 scanner (GE Healthcare). Thoracic kyphosis was quantitatively assessed on lateral radiographs of the same animals using two measurements: kyphotic angles and kyphotic index. Kyphotic angles were calculated by measuring the angle indicated in red on the radiographs. The kyphotic index was calculated from the line between the caudal margin of the last cervical vertebra and the cranial border of the wing of the ilium (line a–b) divided by a line perpendicular to this from the dorsal edge of the vertebra at the point of greatest curvature (line c–d) (Rhodes et al., 2015).

### Statistical analyses

Data are presented as the mean±s.d. Differences between two groups were assessed using unpaired two-tailed Student's *t*-tests. Multiple comparisons between three or more groups were performed using one-way or two-way ANOVA. Statistical differences were considered significant at *P*<0.05 and were calculated using Excel (Microsoft, Redmond, WA, USA) and Prism 8 (GraphPad, La Jolla, CA, USA).

## Supplementary Material

Supplementary information
