# Supplementary material for: A new mouse model of Ehlers-Danlos syndrome generated using CRISPR/Cas9-mediated genomic editing
Source: Dis Model Mech. 2021 Dec 23;14(12):dmm048963. doi: 10.1242/dmm.048963 (PMC8713987; doi:10.1242/dmm.048963)
Supplement: Supplementary information [file dmm-14-048963-s1.pdf]

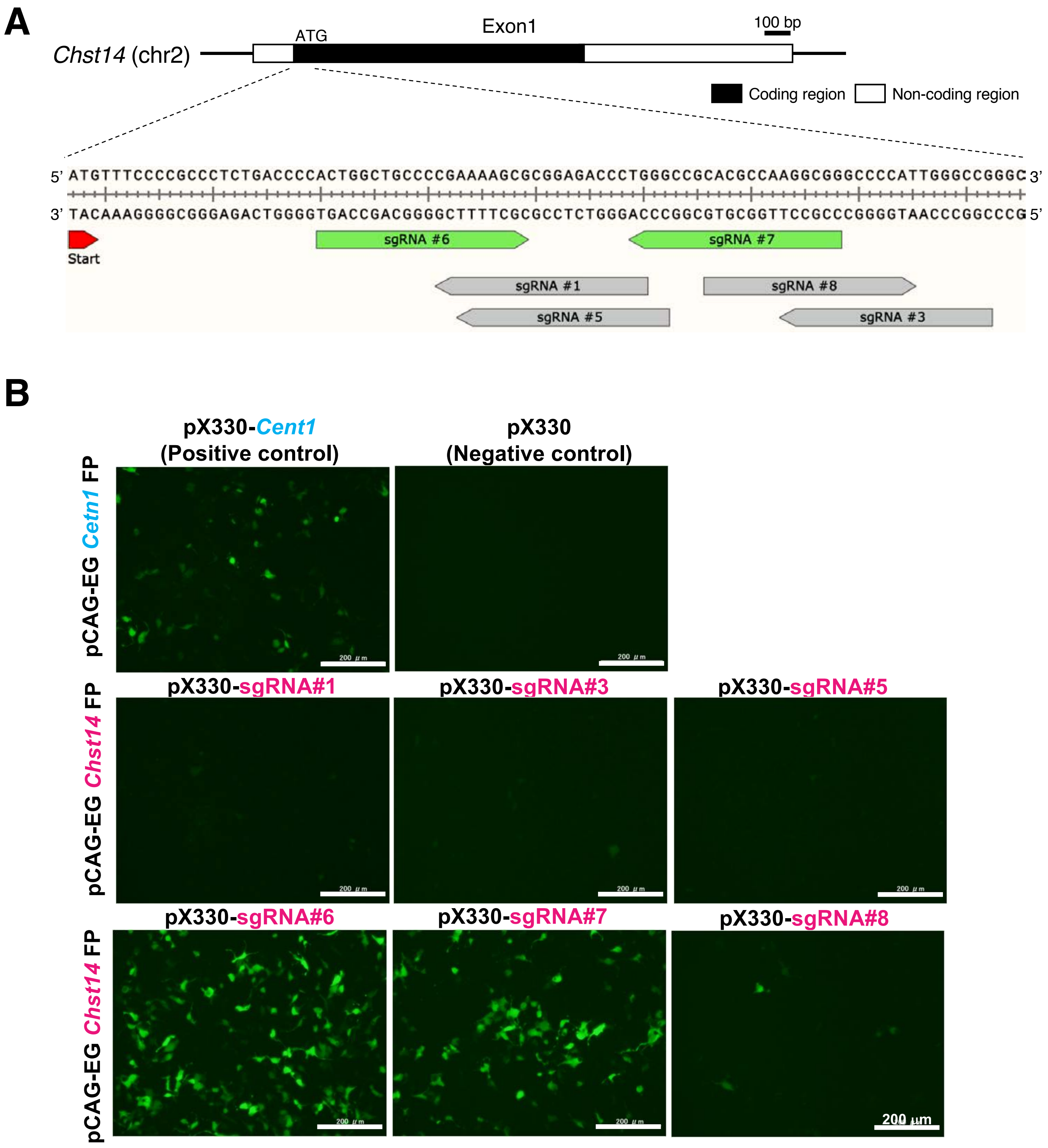

Disease Models & Mechanisms • Supplementary information

Fig. S1. ***In vitro*** **evaluation of the sgRNAs' DNA cleavage activities.**

(A) Six sgRNAs (#1, 3, 5, 6, 7, and 8) to target the downstream region from the *Chst14* translation start site ATG are indicated by arrows within the sequences. (**B**) Screening of candidate sgRNAs using a method that explores homologous recombination and reconstituting EGFP expression. The pCAG-EGxxFP target plasmid contained 5 and 3 EGFP fragments, and a *Chst14* genomic fragment or *Cent1* as a control between EGFP fragments (pCAG-EG *Chst14* FP, or pCAG-EG *Cent1* FP). The target plasmid was co-transfected with pX330 plasmids expressing sgRNA and hCas9 into HEK293T cells. The efficiency of homology-dependent repair was validated by observing EGFP fluorescence 2 days after transfection (pX330 with Cetn1/sgRNA1, positive control; pX330 without sgRNA, negative control). Bars, 200 µm.

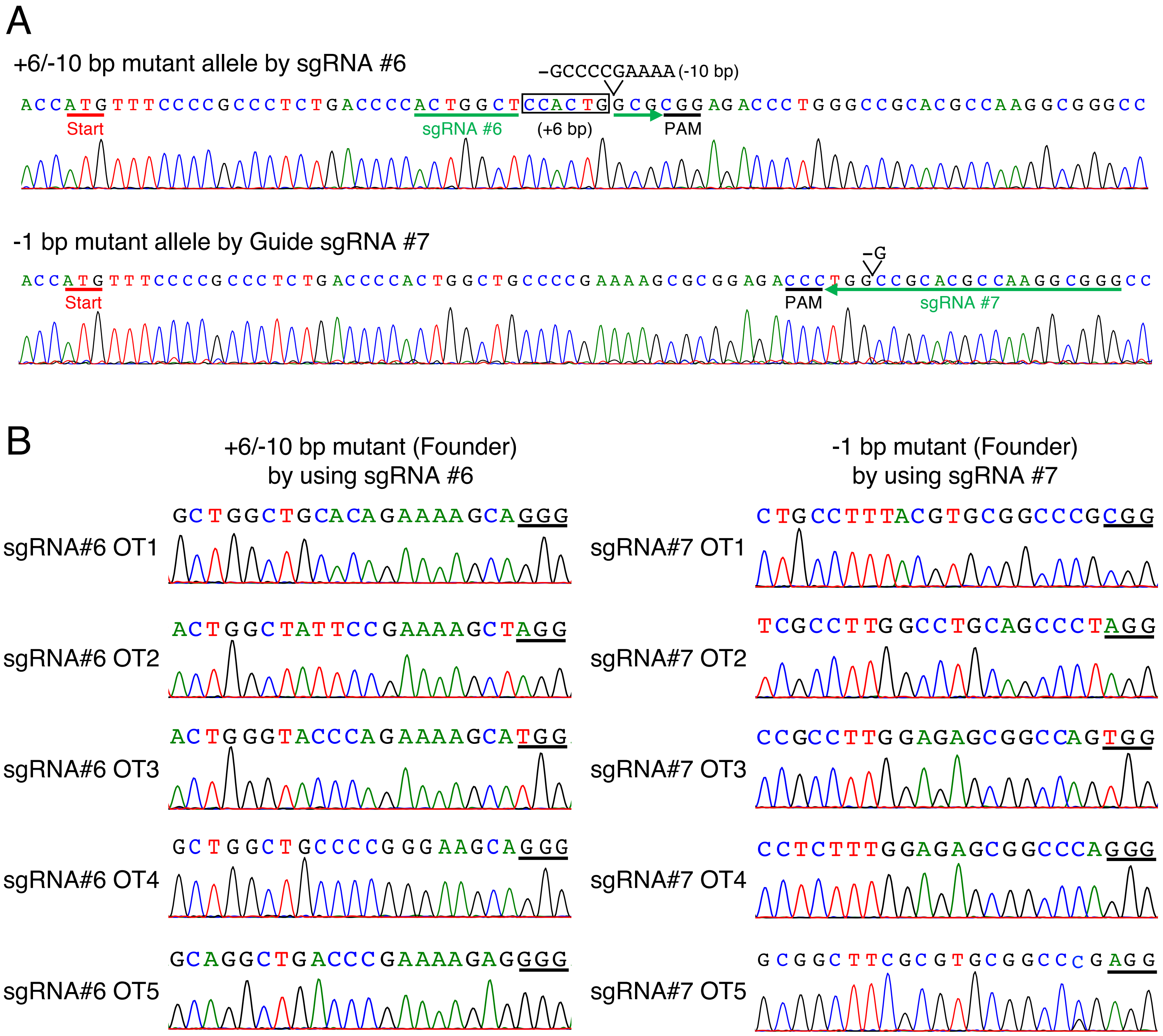

Fig. S2. CRISPR/Cas9-mediated *Chst14* mutant alleles used in this study and their off-target analyses

(A) The detailed sequences for two independent nonsense mutant alleles. **(B)** Sequencing results from five off-target candidate loci for each sgRNAs (#6 and #7) in founder mice. The PAM sequences are underlined. No sign of off-target cleavages was detected within the genomic regions evaluated.

Disease Models & Mechanisms • Supplementary information

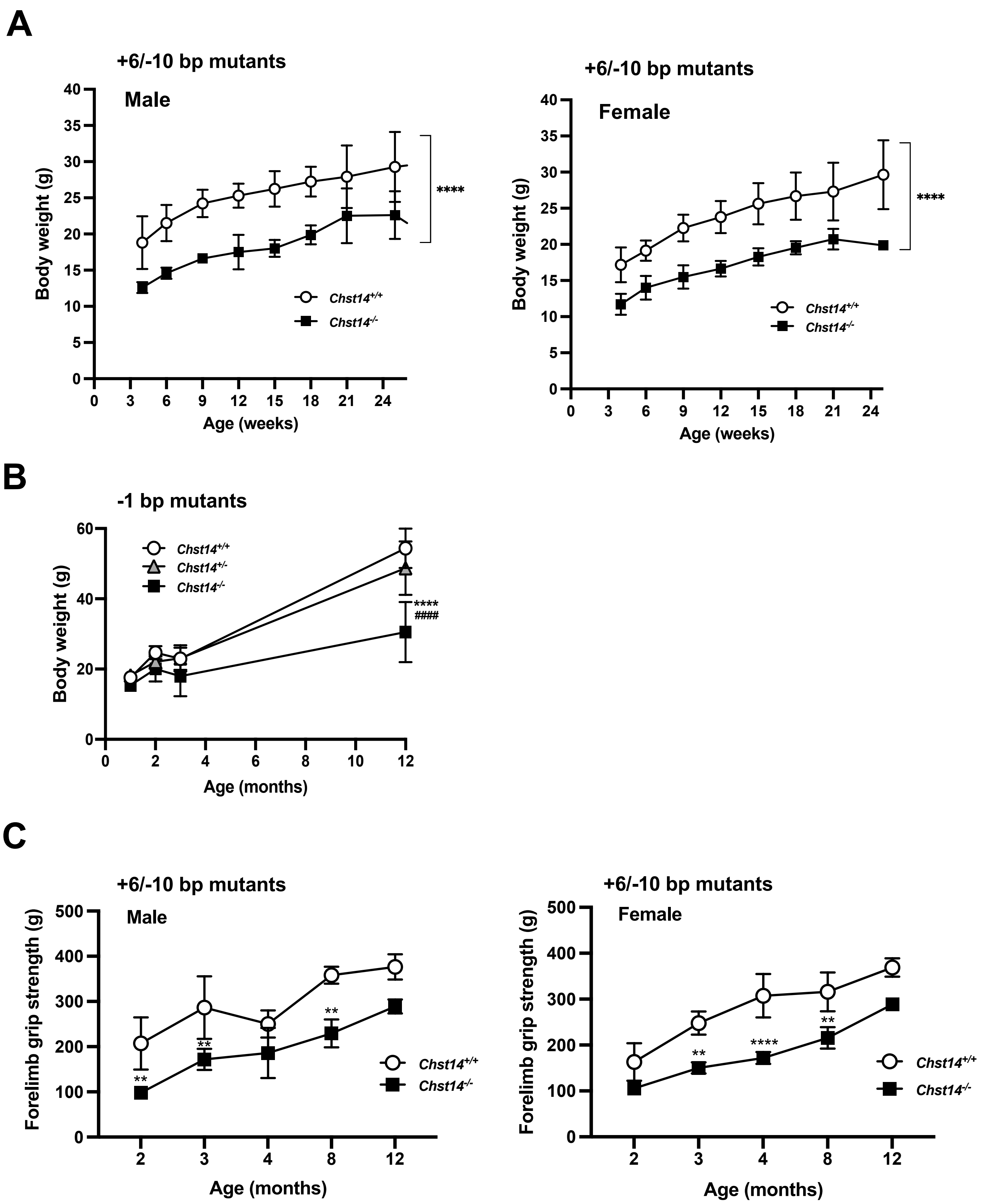
A
+6/-10 bp mutants
Male
Body weight (g)
Age (weeks)
Chst14+/+
Chst14-/-
****
+6/-10 bp mutants
Female
Body weight (g)
Age (weeks)
Chst14+/+
Chst14-/-
****
B
-1 bp mutants
Body weight (g)
Age (months)
Chst14+/+
Chst14+/-
Chst14-/-
****
####
C
+6/-10 bp mutants
Male
Forelimb grip strength (g)
Age (months)
Chst14+/+
Chst14-/-
+6/-10 bp mutants
Female
Forelimb grip strength (g)
Age (months)
Chst14+/+
Chst14-/-

Fig. S3. Reduction in growth curve and grip strength in *Chst14* mutant mice

(**A**) Growth curve of 1- to 24-week-old male and female *Chst14*$^{+/+}$ ($n = 3$−4), and +6/−10-bp mutant homozygous mice (*Chst14*$^{-/-}$, $n = 3$–4). (**B**) Growth curve of 1- to 12-month-old *Chst14*$^{+/+}$ ($n = 5$–8), and −1-bp mutant heterozygous (*Chst14*$^{+/-}$, $n = 6$–9) and homozygous mice (*Chst14*$^{-/-}$, $n = 3$–4). (**C**) Grip strength data (g) from 2- to 12-month-old male and female *Chst14*$^{+/+}$ ($n = 3$), and *Chst14*$^{-/-}$ mutant mice (+6/−10-bp mutant, $n = 3$). All data are presented as mean ± SD. Significant differences, *Chst14*$^{+/+}$ vs. *Chst14*$^{-/-}$ ($^{**}p < 0.005$; $^{****}p < 0.0001$), *Chst14*$^{+/-}$ vs. *Chst14*$^{-/-}$ ($^{\#\#\#\#}p < 0.0001$), were evaluated using two-way ANOVA, and *t*-test.

Disease Models & Mechanisms • Supplementary information

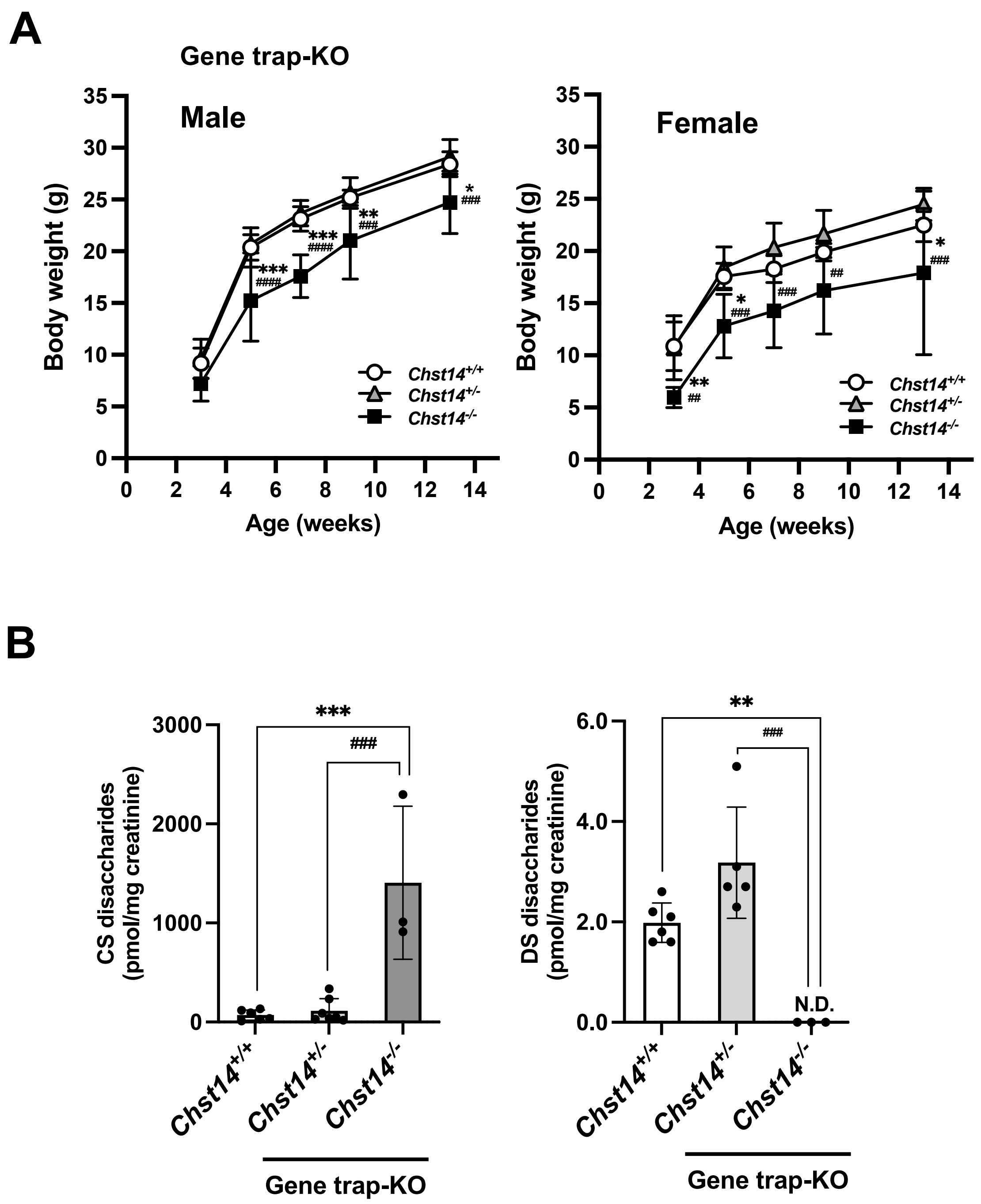
A
Gene trap-KO
Male
Female
Body weight (g)
Age (weeks)
Chst14+/+
Chst14+/-
Chst14-/-
B
CS disaccharides
(pmol/mg creatinine)
DS disaccharides
(pmol/mg creatinine)
N.D.
Gene trap-KO
Chst14+/+
Chst14+/-
Chst14-/-

Fig. S4. Early-life growth rate and deficiency of dermatan sulfate biosynthesis in *Chst14* gene trap-knockout (KO) mice

(**A**) Early-life growth rate of male and female *Chst14*$^{+/+}$ (n = 5), gene trap-KO *Chst14*$^{+/-}$ (n = 9), and *Chst14*$^{-/-}$ mice (n = 3). (**B**) Total amounts of chondroitin sulfate (CS) anddermatan sulfate (DS) disaccharides derived from urine of 5−6-week-old *Chst14*$^{+/+}$ (n = 6), *Chst14*$^{+/-}$ (n = 5), and *Chst14*$^{-/-}$ mice (n = 3) were analyzed by anion-exchange HPLC after enzymatic digestion. N.D., not detected (< 0.1 pmol/mg creatinine). All data are presented as mean ± SD. Significant differences, *Chst14*$^{+/+}$ vs. *Chst14*$^{-/-}$ ($^{*}p$ < 0.05, $^{**}p$ < 0.01, $^{***}p$ < 0.001, and $^{****}p$ < 0.0001), *Chst14*$^{+/-}$ vs. *Chst14*$^{-/-}$ ($^{\#\#}p$ < 0.01, $^{\#\#\#}p$ < 0.001, $^{\#\#\#\#}p$ < 0.0001), were evaluated using two-way or one-way ANOVA.

Disease Models & Mechanisms • Supplementary information

A

CSase AC digests of GAGs

−1 bp mutants

Chst14$^{+/+}$ ***Chst14***$^{+/-}$ ***Chst14***$^{-/-}$

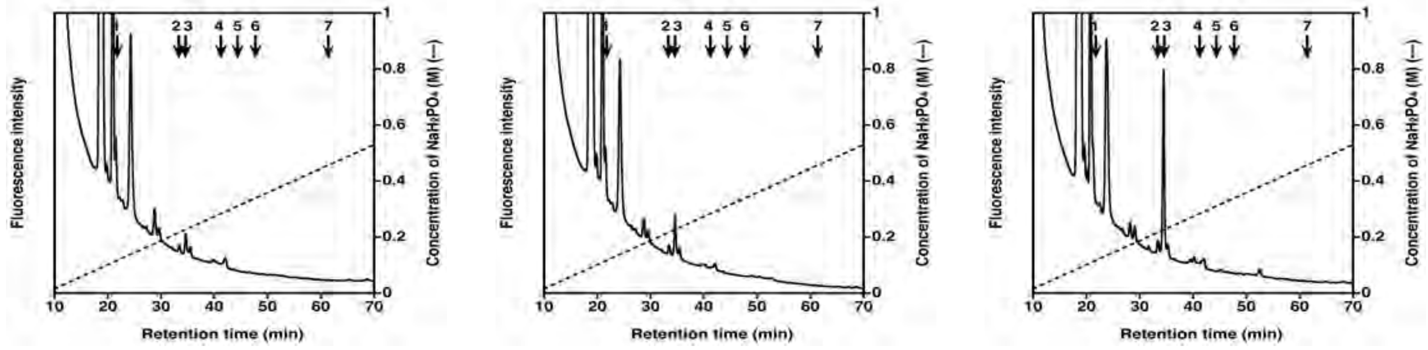

B

CSase B digests of GAGs

−1 bp mutants

Chst14$^{+/+}$ ***Chst14***$^{+/-}$ ***Chst14***$^{-/-}$

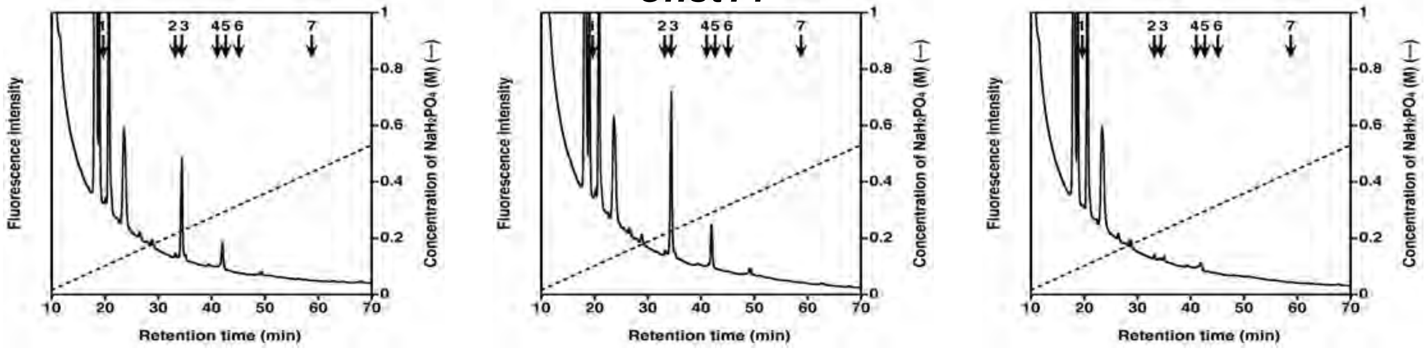

Fig. S5. Chromatograms of the chondroitinase AC and B digests of glycosaminoglycans derived from muscles

Chromatograms show chondroitinase AC (**A**) and chondroitinase B (**B**) digests of glycosaminoglycans derived from *Chst14*$^{+/+}$, −1-bp mutant *Chst14*$^{+/-}$, and *Chst14*$^{-/-}$ mice. The elution positions of authentic 2-aminobenzamide-labeled CS/DS disaccharides are indicated by the following numbers: 1, ΔHexUA-GalNAc; 2, ΔHexUA-GalNAc(6S); 3, ΔHexUA-GalNAc(4S); 4, ΔHexUA(2S)-GalNAc(6S); 5, ΔHexUA(2S)-GalNAc(4S); 6, ΔHexUA GalNAc(4S,6S); 7, ΔHexUA(2S)-GalNAc(4S,6S). ΔHexUA, GalNAc, 2S, 4S, and 6S represent 4,5-unsaturated hexuronic acid, *N*-acetylgalactosamine, 2-*O*-sulfate, 4-*O*-sulfate, and 6-*O*-sulfate, respectively.

Disease Models & Mechanisms • Supplementary information

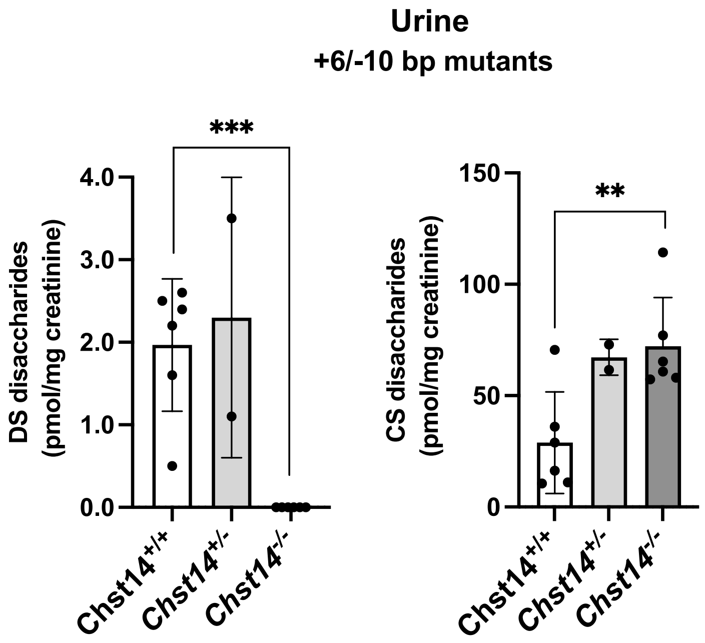

Fig. S6. Total amounts of CS and DS disaccharides derived from urine

The total amounts of CS and DS disaccharides derived from urine of 1-y ear-old *Chst14*$^{+/+}$, +6/−10-bp mutants *Chst14*$^{+/-}$, and *Chst14*$^{-/-}$ mutant female mice were analyzed by anion-exchange HPLC after enzymatic digestion. Data are presented as mean ± SD. Statistical differences, compared to *Chst14*$^{+/+}$ mice and *Chst14*$^{-/-}$ mutant mice (n = 6; $^{**}p < 0.01$, $^{***}p < 0.001$), were evaluated using t-test; N.D., not detected (< 0.1 pmol/mg creatinine).

Disease Models & Mechanisms • Supplementary information

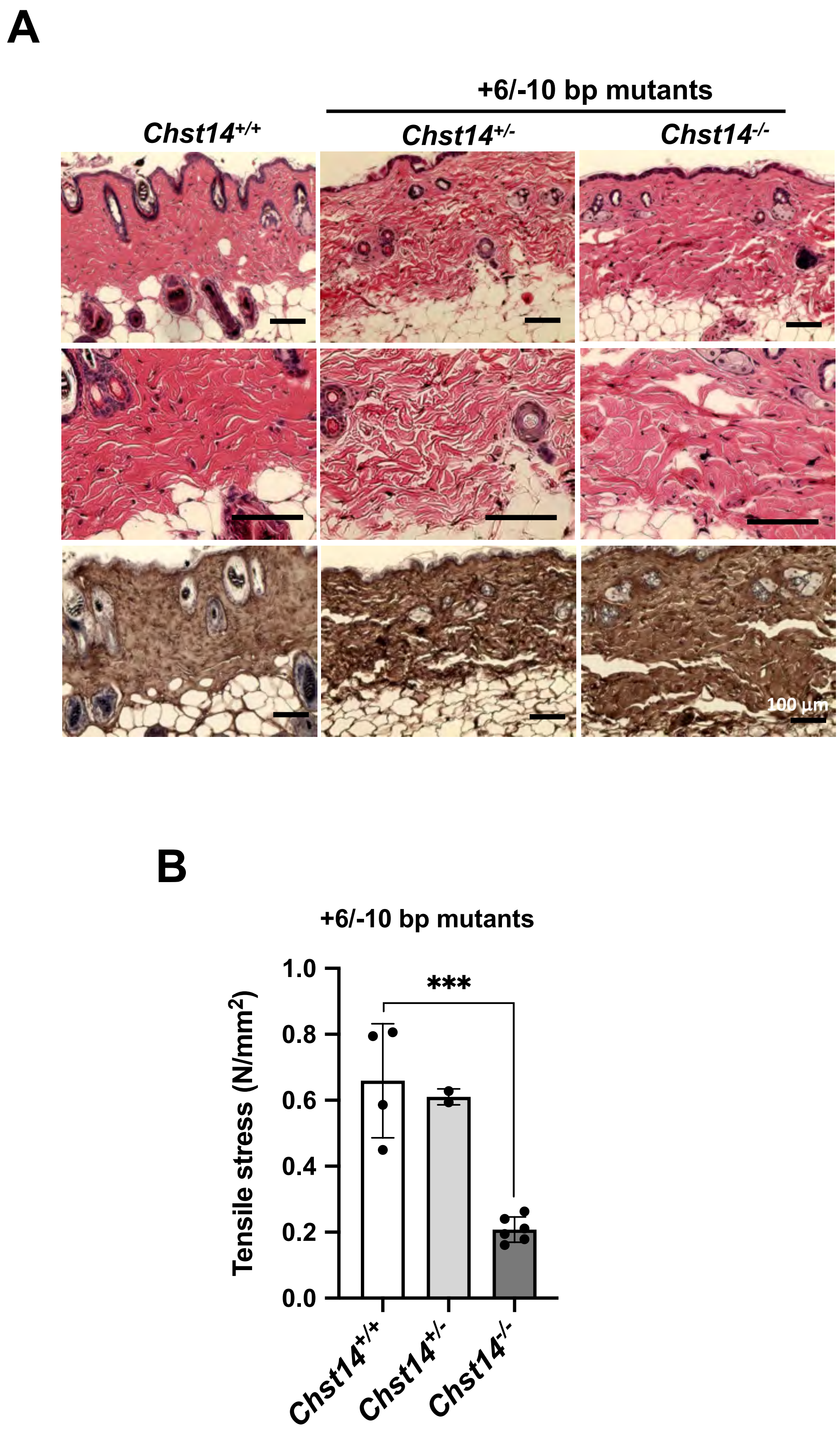

Fig. S7. Skin fragility in *Chst14* mutant mice

(**A**) Hematoxylin and eosin (H&E) staining (upper and middle panels) and immunohistochemical analysis by horseradish peroxidase (HRP)-diaminobenzidene

(DAB)-labeled observation of decorin (bottom panels) in skin derived from *Chst14*$^{+/+}$, +6/−10-bp mutant *Chst14*$^{+/-}$, and *Chst14*$^{-/-}$ mice. Bars, 100 µm. (**B**) Tensile stress (N/mm^2) were derived from *Chst14*$^{+/+}$, +6/−10-bp mutants *Chst14*$^{+/-}$, and *Chst14*$^{-/-}$ mice. Data are presented as mean ± SD. Statistical differences, compared to *Chst14*$^{+/+}$ ($^{***}p < 0.001$) were evaluated using one-way ANOVA.

Disease Models & Mechanisms • Supplementary information

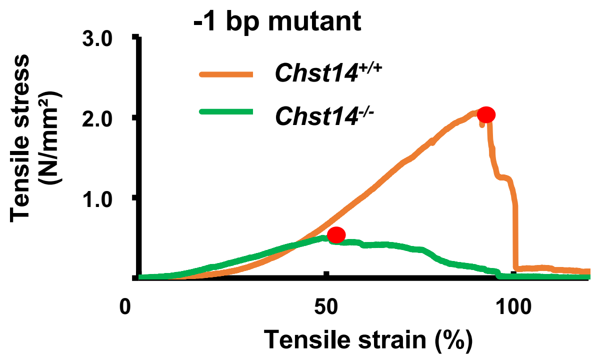

Fig. S8. Tensile stress and stress-strain curves from the skin of *Chst14* mutant mice The tensile stress and tensile strain on the skin were derived from the linear slopes of the force-displacement and stress-strain curves for *Chst14*$^{+/+}$ and −1-bp mutant *Chst14*$^{-/-}$ mice, respectively. Red dots indicate the points at the maximum stress.

Disease Models & Mechanisms • Supplementary information

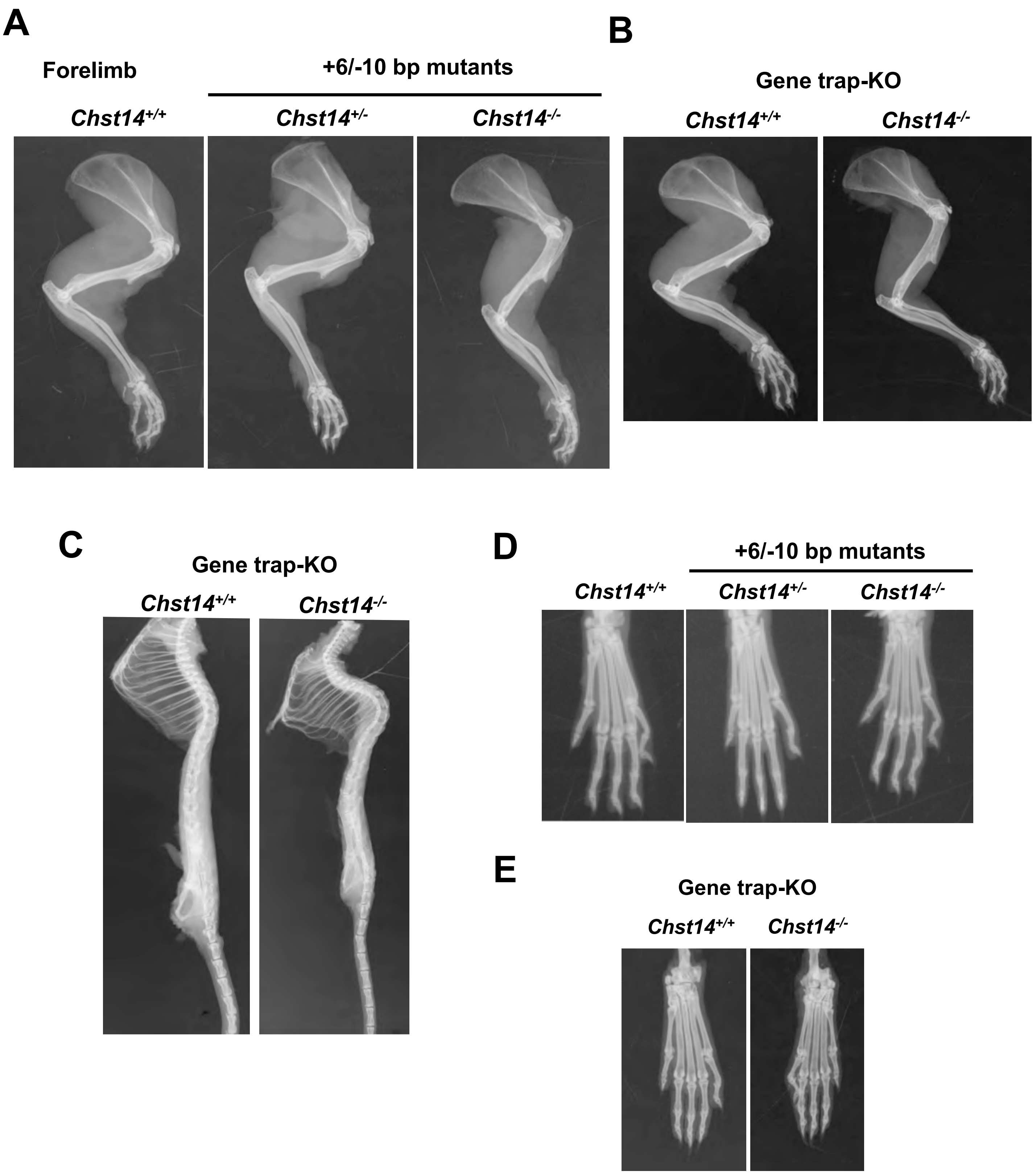

Fig. S9. Radiographs of *Chst14* mutant mice

(**A, B**) Forelimb radiographs for 1-year-old female *Chst14*$^{+/+}$, +6/−10-bp mutants *Chst14*$^{+/-}$, and *Chst14*$^{-/-}$ mice (**A**), and *Chst14* gene trap-KO mice (**B**). (**C**) Representative lateral radiographs of 1-year-old female *Chst14*$^{+/+}$ and *Chst14*$^{-/-}$ gene trap-KO mice. (**D, E**) Hindlimb radiographs of 1-year-old female *Chst14*$^{+/+}$, +6/−10-bp mutants *Chst14*$^{+/-}$, and *Chst14*$^{-/-}$ (**D**), and *Chst14* gene trap-female KO mice (**E**).

A

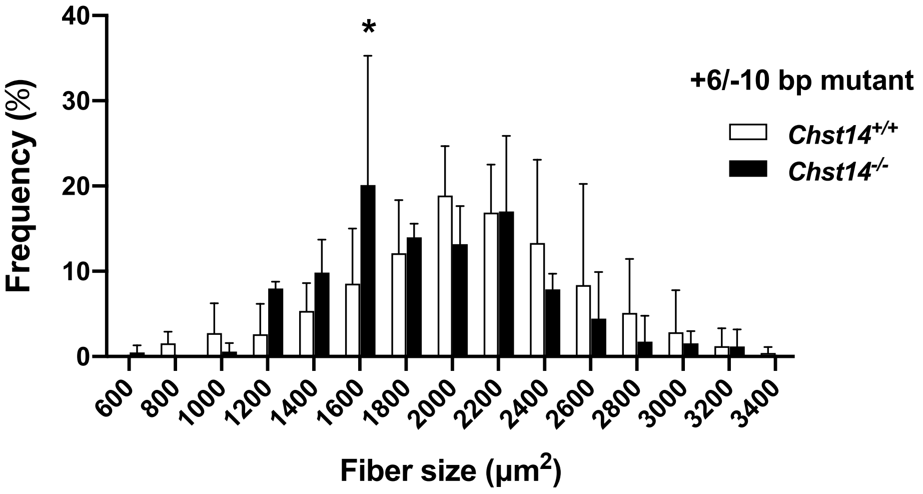

B

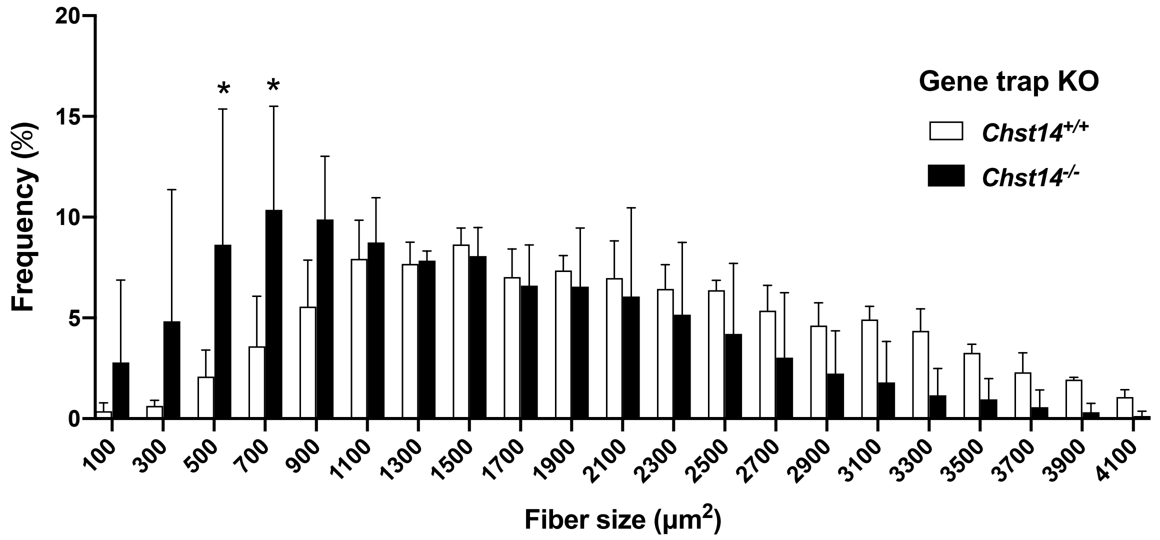

Fig. S10. The frequency distribution of myofiber area in *Chst14* mutant mice and gene trap-KO-mouse

Frequency distribution of myofiber area (µm^2) counted from H&E staining photographs of 1-year-old *Chst14*$^{+/+}$, +6/−10-bp mutant *Chst14*$^{-/-}$ mice (**A**) and *Chst14* gene trap-KO mice (**B**) (n = 3, each). Area values show frequency (% of total fibers) and distribution comparisons. Data are represented as mean ± SD. Significant differences compared to *Chst14*$^{+/+}$ ($^*p < 0.05$) were evaluated using the multiple t-test.

Disease Models & Mechanisms • Supplementary information

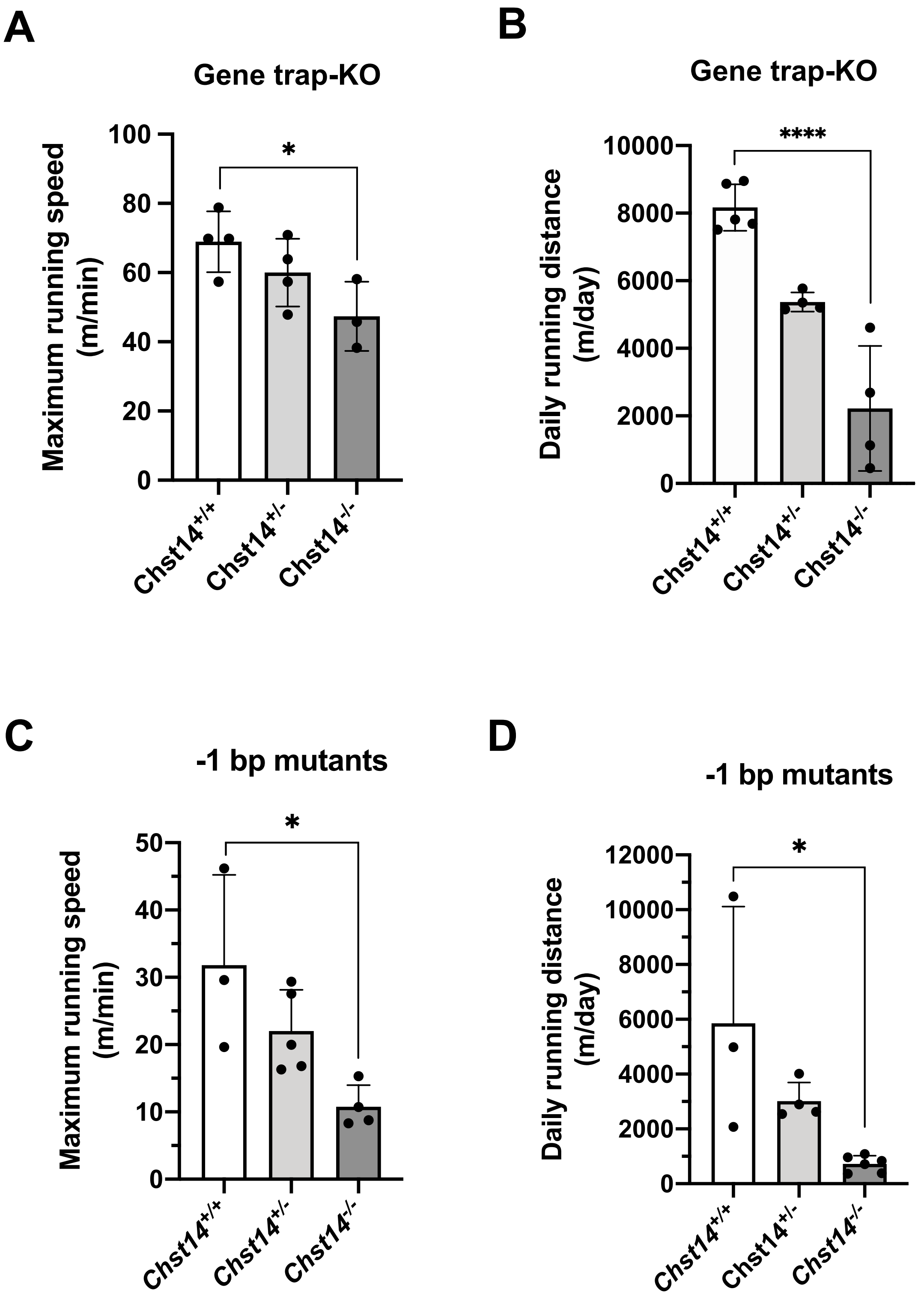

Fig. S11. Reduced voluntary activity in *Chst14* mutant mice and gene trap-KO mice The mice were analyzed for voluntary running activity in the wheel cage, yielding voluntary running activity and maximum running speed (m/min) (**A, C**), and the daily running distance (**B, D**) for 1-year-old *Chst14*$^{+/+}$ (n =3–4), *Chst14*$^{+/-}$ (n =4), and *Chst14*$^{-/-}$ gene trap-KO mice (n = 3) (**A, B**), and −1-bp mutant mice (n = 3–4) (**C, D**). All data are presented as the mean ± SD. Significant differences compared to *Chst14*$^{+/+}$ ($^{*}p < 0.05$, and $^{****}p < 0.0001$) were evaluated using one-way ANOVA.

Disease Models & Mechanisms • Supplementary information

Table S1. Substitution rate of congenic marker

To discriminate 129S2/SvImJ and C57BL/6J mice strain-specific 58 SSLP markers, DNA from mouse tails was amplified by PCR using SSLP-specific primers. Heterozygous mice with the highest replacement rate for markers of 129 mice in B6 mice were selected for mating to obtain the next generation. The tables show the numbers of C57BL/6J marker and heterozygous 129S2/SvImJ and C57BL/6J markers and their substitution rate for C57BL/6J (%) in F9 and F11 generation gene trap mice (total 8 or 7 mice, respectively).

F9 generation gene trap mouse #	Marker (N° of sites)		Substitution rate (%)
	C57BL/6J	129S2/SvImJ x C57BL/6J	
1	33	11	75.0
2	40	18	69.0
3	42	16	72.4
4	48	10	82.8
5	42	16	72.4
6	42	15	73.7
7	42	14	75.0
8	39	18	68.4

F11 generation gene trap mouse #	Marker (N° of sites)		Substitution rate (%)
	C57BL/6J	129S2/SvImJ x C57BL/6J	
1	55	3	94.8
2	53	5	91.4
3	55	3	94.8
4	55	3	94.8
5	56	2	96.6
6	55	3	94.8
7	57	1	98.3

Table S2. Birth rate of gene trap *Chst14* KO mice

Birth (Number, Rate %)			
Chst14$^{+/+}$	*Chst14*$^{+/-}$	*Chst14*$^{-/-}$	Birth (Total)
78 (25.7 %)	220 (72.6 %)	5 (1.7 %)	303 (100%)

Table S3. Offspring number of CRISPR/Cas9 genomic-engineered mice

Genotype	Newborn mouse numbers	
	sgRNA #6	sgRNA #7
Homozygous (Frameshift mutation*)	1	1
Heterozygous	1	0
Compound heterozygous	18	19
Wild type	0	1
Total	20	21

*, The mutation was introduced a premature termination codon.

Disease Models & Mechanisms • Supplementary information

Table S4. A. The potential off-target candidate loci (OT1-OT5) for *Chst14* sgRNA #6 and #7 predicted by CRISPOR web-tool.

		Off-target sequence	Mismatch position	Mismatch count	MIT Off-target score	CFD Off-target score	Chromosome	Start	End	Strand	Locus description
**sgRNA #6**	OT1	GCTGGCTGCACAGAAAAGCAGGG	*........*.*.......*	4	0.316	0.580	chr17	46562060	46562082	+	intergenic:Srf-Ptk7
	OT2	ACTGGCTATTCCGAAAAGCTAGG	.......***.........*	4	0.353	0.576	chr7	79968057	79968079	+	intergenic:Gm24541-Zfp710
	OT3	ACTGGGTACCCAGAAAAGCATGG	.....*.*...*.......*	4	0.187	0.335	chr11	76104879	76104901	+	intron:Vps53
	OT4	GCTGGCTGCCCCGGGAAGCAGGG	*.........**....*	4	0.028	0.284	chr17	87382553	87382575	-	intergenic:Ttc7-1700011E24Rik
	OT5	GCAGGCTGACCCGAAAAGAGGGG	*.*.....*.........*.	4	0.317	0.262	chr1	176317840	176317862	+	intergenic:Pld5-Gm26104
**sgRNA #7**	OT1	CTGCCTTTACGTGCGGCCCGCGG	.*.....**..........*	4	0.426	0.279	chr11	103360459	103360481	-	exon:Arhgap27/Gm11647
	OT2	TCGCCTTGGCCTGCAGCCCTAGG	*.........*...*....*	4	0.104	0.242	chr12	69184554	69184576	-	exon:Mgat2
	OT3	CCGCCTTGGAGAGCGGCCAGTGG	.........*.*......**	4	0.085	0.227	chr19	43028047	43028069	-	intergenic:Hpse2-Gm22135
	OT4	CCTCTTTGGAGAGCGGCCCAGGG	..*.*....*.*........	4	0.639	0.221	chr14	25353987	25354009	+	intergenic:Gm10398-Gm26660
	OT5	GCGGCTTCGCGTGCGGCCCGAGG	*..*...*...........*	4	0.697	0.215	chr14	56886933	56886955	+	intergenic:Gm22218-Zmym2

On target sequence for sgRNA #6; ACTGGCTGCCCCGAAAAGCG CGG (chr14) On target sequence for sgRNA #7; CCGCCTTGGCGTGCGGCCCA GGG (chr14)

Disease Models & Mechanisms • Supplementary information

B. Primers used for Off-target analyses

Name	Sequence (5' to 3')
sgRNA #6 OT1_F	AGAAGCTCTTAGCAGTGATGGCAG
sgRNA #6 OT1_R	GTCTTGAACTCGCTACAGAGCTAAGG
sgRNA #6 OT2_F	CCTGCACCTAGTGACAGGTAGTGATTC
sgRNA #6 OT2_R	ATCTGCAAGGTTTGTATACTCCTCAGC
sgRNA #6 OT3_F	CAGAGAGTGTTCCTGGAGTCTATGTCC
sgRNA #6 OT3_R	TTGCATGGCAAGTGAATGCTGTTG
sgRNA #6 OT4_F	GATGACTGGAGCACATACAGGTGG
sgRNA #6 OT4_R	GTGGTAAAGGGAACATTCTAGCCATCC
sgRNA #6 OT5_F	GAAGTCATCAGGACTGCACAGCAATC
sgRNA #6 OT5_R	GGTCTGCATTCTCAGAACACCCAC
sgRNA #7 OT1_F	TAGACTCTGCTGGACTGTGACCTC
sgRNA #7 OT1_R	TACGACTACCGCTTCGTGAGCAC
sgRNA #7 OT2_F	CGCAGGTAAAGGAATCACGGTGTC
sgRNA #7 OT2_R	CATTGCTACTCCAGAGGACGAAGC
sgRNA #7 OT3_F	CATGCGCTGGAAAGTCAAAGTAGC
sgRNA #7 OT3_R	CATACGTACACTGAGTGCCTTCCTG
sgRNA #7 OT4_F	CTAGTCACCTGTGAAAGTGAACCTGAG
sgRNA #7 OT4_R	AGTCTAGCAGGCACATGAGATTCCTC
sgRNA #7 OT5_F	CAGAACCCGGTGAGGCTTCTC
sgRNA #7 OT5_R	TCTCGGTTGGACAGACAGGGTG

Disease Models & Mechanisms • Supplementary information
